# Macroaggregates Serve as Micro-Hotspots Enriched With Functional and Networked Microbial Communities and Enhanced Under Organic/Inorganic Fertilization in a Paddy Topsoil From Southeastern China

**DOI:** 10.3389/fmicb.2022.831746

**Published:** 2022-04-11

**Authors:** Zhipeng Rui, Xinda Lu, Zichuan Li, Zhi Lin, Haifei Lu, Dengxiao Zhang, Shengyuan Shen, Xiaoyu Liu, Jufeng Zheng, Marios Drosos, Kun Cheng, Rongjun Bian, Xuhui Zhang, Lianqing Li, Genxing Pan

**Affiliations:** ^1^Institute of Resource, Ecosystem and Environment of Agriculture, Nanjing Agricultural University, Nanjing, China; ^2^Department of Soil Science, Nanjing Agricultural University, Nanjing, China; ^3^Jiangsu Collaborative Innovation Center for Solid Organic Waste Resource Utilization, Nanjing Agricultural University, Nanjing, China; ^4^Department of Civil and Environmental Engineering, Massachusetts Institute of Technology, Cambridge, MA, United States; ^5^Center of Agro-Technology Extension, Suzhou, China

**Keywords:** rice paddy, aggregate-size fraction, microbial community, soil organic carbon, co-occurrence network, functional profile

## Abstract

Microbial communities of soil aggregate-size fractions were explored with molecular and networking assays for topsoil samples from a clayey rice paddy under long-term fertilization treatments. The treatments included no fertilizer (NF) as control, chemical fertilizer only (CF), chemical fertilizer with swine manure (CFM), and chemical fertilizer with rice straw return (CFS). Following a wet-sieving protocol, water-stable aggregates were separated into size fractions of large macroaggregates (L-MacA, >2,000 μm), macroaggregates (MacA, 2,000–250 μm), microaggregates (MicA, 250–53 μm), fine microaggregates (F-MicA, 53–2 μm), and fine clay (F-Clay, <2 μm). Mass proportion was 32.3–38.2% for F-MicA, 23.0–31.5% for MacA, 19.0–23.1% for MicA, 9.1–12.0% for L-MacA, and 4.9–7.5% for F-Clay, respectively. The proportion of MacA was increased, but F-Clay was reduced by fertilization, whereas the mean weight diameter was increased by 8.0–16.2% from 534.8 μm under NF to 621.5 μm under CFM. Fertilization affected bacterial 16S rRNA and fungal 18S rRNA gene abundance in F-MicA and F-Clay but not in aggregates in size larger than 53 μm. However, bacterial and fungal community α-diversities and community structures were quite more divergent among the fertilization treatments in all size fractions. Organic carbon and gene abundance of bacteria and fungi were enriched in both L-MacA and MacA but depleted in F-Clay, whereas microbial Shannon diversity was rarely changed by fraction size under the four treatments. L-MacA and MacA contained more bacteria of r-strategists and copiotrophs, whereas F-MicA and F-Clay were demonstrated with a higher abundance of K-strategists and oligotrophs. Guilds of parasitic and litter saprotrophic fungi were enriched in F-MicA but depleted in L-MacA. Furthermore, most of bacterial and fungal operational taxonomic units were strongly interacted in L-MacA and MacA rather than in MicA and F-Clay. Thus, MacA acted as micro-hotspots enriched with functional and networked microbial communities, which were enhanced with organic/inorganic fertilization in the rice paddy.

## Introduction

Soil structure is known to be essential for soil porosity, water movement, solute transport, gas exchanges, carbon sequestration (Six et al., 2002; Pituello et al., 2016), and biological components (Tecon and Or, 2017). Soil aggregation, a key soil formation process, can greatly affect soil biophysical entity, resulting in distinct microbial communities (Wilpiszeski et al., 2019) and consequent biogeochemical processes (Lehmann and Rillig, 2015). Meanwhile, soil microbial biota in turn can create protected microcosms within soil and mediate microscale ecological interactions (Vos et al., 2013). Microbial populations are spatially isolated and stabilized within soil aggregates, often regarded as “concurrent incubators” for the evolution of microbial communities inside (Rillig et al., 2017). Therefore, variations in composition and function of microbial communities across aggregate-size fractions are increasingly addressed as microscale mechanisms in soil biogeochemical processes, owing to the controls on overall performance and the mediation of element cycling in soil (Six et al., 2000; Rillig et al., 2017; Li et al., 2019c; Wilpiszeski et al., 2019).

Soil organic matter (SOM) plays a vital role in maintaining global carbon balance, supporting food production and sustaining soil ecosystem services (Lal, 2004; Powlson et al., 2011; Schmidt et al., 2011; Wiesmeier et al., 2019). Soil microorganisms, the key driver of SOM turnover (Gleixner, 2013; Kallenbach et al., 2016), can also be largely shaped by SOM in reverse in community structure (Roller and Schmidt, 2015). SOM accumulation is often associated with distinct microbial communities with higher fungal abundances and efficient microbial biomass production (Kallenbach et al., 2016). Furthermore, soil microhabitats with higher labile organic content and microbial population higher in carbon metabolism efficiency are suggested as microbial hotspots for C biogeochemical cycling in soil, compared with bulk soil (Kuzyakov and Blagodatskaya, 2015). The distribution, decomposability, and accessibility of SOM, at both spatial and temporal scales, substantially determine the microbial and functional diversity among aggregate-size fractions (Marx et al., 2005; Wallenius et al., 2011; Lagomarsino et al., 2012). In response to SOM accumulation, changes in microbial activity can cope with stabilizing organic carbon and soil aggregates for ecological functioning (Tecon and Or, 2017). For example, microbial metabolites can contribute notably to aggregate stabilization by binding mineral particles and/or microaggregates to form macroaggregates (MacA) (Wander, 2004), within which particulate organic matter (POM) is preserved and physically protected by spatially excluding from microbes and enzymes (Six et al., 2000; Martens et al., 2004; Lützow et al., 2006; Six and Paustian, 2014). As a major C sequestration mechanism, the extent of microbes accessing to SOM under physical protection can be conferred by localization of microbial communities, extracellular enzyme activities, and carbon substrates among soil aggregates (Dungait et al., 2012; Navarro-Garcia et al., 2012; Trivedi et al., 2017). Therefore, in agricultural management, organic fertilization is the most effective way to improve soil fertility by promoting SOM content, soil aggregation, and microbial activity (Zhang et al., 2007, 2015; Wang et al., 2017; Li et al., 2019c).

Traditionally, we often research soil functions especially related to C and N cycling by determining the specific extracellular enzyme activities. For example, our previous work has addressed that carbon-degrading enzymes and N-acetylglucosaminidase activities are enriched in >53-μm aggregate fractions under different fertilization, whereas β-glucosidase and cellobiohydrolase activities are enhanced under CFS (Li et al., 2019c). However, we cannot achieve a general view of soil functioning mediated by microbial community in detail in such manner. Recently, the tools of PICRUSt (Phylogenetic Investigation of Communities by Reconstruction of Unobserved States) and FUNGuild (Fungi functional guild) have been increasingly used for researching the functional profiles of bacterial (Hou et al., 2019; Wang et al., 2019; Liu et al., 2020; Lu et al., 2020; Ma et al., 2020) and fungal community (Schlatter et al., 2018; Li et al., 2019b; Lu et al., 2020), respectively, based on amplicon sequencing in soil science. By analyzing functional results, the general metabolic functions of soil microbial community can also be linked to microbial trophic and ecological strategy (Koch, 2001; Lauro et al., 2009; Song et al., 2017). For instance, Song et al. (2017) addressed that a high (copiotrophic) or low (oligotrophic) nutrient strategy was rich in genes, respectively, related to cell division and cell cycle or related to carbohydrate metabolism and virulence, disease, and defense, whereas an r- or K-strategy was rich in genes, respectively, related to regulation and cell signaling or related to motility and chemotaxis, whereas these tools have been rarely applied to studying microbial functional profiles of communities across soil aggregate-size fractions. At the macroscale, soil microbial interactions are known to be altered by abiotic or biotic factors, or both, which can also result in specific community structure and metabolic functional profile (Prosser et al., 2007; Barberan et al., 2012; Ma et al., 2016; Schlatter et al., 2018; Schmid et al., 2018; Hou et al., 2019). Using high-throughput sequencing data sets, network analysis of significant taxon co-occurrence patterns of soil microbiota can help to disentangle community assembly patterns and interactions among microbial individuals within microbial consortia (Horner-Devine et al., 2007; Fuhrman, 2009). Meanwhile, based on the analysis of co-occurrence network, bacterial functional complexity and community interactions also have been used for demonstrating geographical variation of soil microbiome (Ma et al., 2016) and for assessing the associations between microbial interaction and soil quality (Schmid et al., 2018).

Paddy soil, as a unique type of anthropogenic soil formed due to long-term hydragric management for rice production under prolonged surface flooding (Gong et al., 2007), has higher SOC storage and sequestration potential compared with dry croplands (Wu et al., 2003; Pan et al., 2004; Haefele et al., 2014). Long-term rice cultivation leads to an accumulation of SOM and in turn soil microbial abundance and activity, with the latter positively correlated to POM accumulation in MacA (Wang et al., 2015; Liu et al., 2016). Optimized management practices including combined organic/inorganic fertilization can enhance SOM accumulation and microbial activity, thus improving soil fertility of paddy soils (Li et al., 2007; Pan et al., 2009). With long-term field experiments, soil macroaggregation of rice paddy has been seen significantly enhanced under organic/inorganic fertilization compared with mineral fertilization (Zhang et al., 2014; Wang et al., 2018). Under combined application of rice straw and balanced chemical fertilizers (Ding et al., 2018), gene abundance and diversity of microbes involved in carbon and nitrogen cycling are increased, and the overall community shifts toward better nutrient biotransformation and plant productivity.

However, the comprehensive and deep study to disentangle how soil performs ecosystem service functions through microbial functioning in different-size fractions of aggregates under different fertilization is still limited. The knowledge of microbial community interactions and functions at the aggregate scale is still insufficient for understanding soil processes governed by soil microbes (Wilpiszeski et al., 2019). Moreover, the distribution of microbial community and functional groups and how they coexist or network within aggregates sustaining carbon and nitrogen cycling in rice paddy soil under long-term fertilization remain yet unclear.

Therefore, it was hypothesized that microbial abundance, diversity, and functional association concerning C cycling could be enhanced in MacA of fertilized paddy soil, due to a physically protected labile C pool and diverse mineral-associated stable C pool in microaggregates within MacA. We also hypothesized that long-term organic/inorganic fertilization could promote the microbial diversity and co-occurrence networks among the water-stable aggregates of rice paddy, by increasing soil macroaggregation. Taking a paddy soil under long-term conventional fertilization as a case, this study aims to provide novel knowledge of microbial communities, interactions, and functions among aggregate-size fractions for understanding SOM accumulation and ecosystem functioning improvement under rational soil management to sustain rice agriculture.

## Materials and Methods

### Experiment Site and Soil

In this study, topsoil samples from a rice paddy were used under a long-term different fertilizer application trial initiated in 1987. The experiment site was located in Jinjiaba Township (31°12′15″ N, 120°15′15″ E), Suzhou Municipality, Jiangsu Province, China. Lying in the central Tai Lake plain, the area was controlled by a humid subtropical monsoon climate, with a mean annual temperature of 15.9°C and precipitation of 1,110 mm, during 1993–2013. Derived from paleo-alluvial/lacustrine deposit, because of the long history of hydroagric management for rice production, the soil had developed as an Entic Halpudept (Soil Survey Staff, 2014) or a Ferric-accumulic Stagnic Anthrosol according to Chinese Soil Taxonomy (Gong et al., 2007). The soil had been conventionally cultivated with summer rice (*Oryza sativa* L.) and winter rape (*Brassica campestris* L.) in an annual rotation for the last decades. The soil texture was clayey loam with 30.3% of clay (<2 μm). The basic properties of topsoil before the experiment were soil pH (H_2_O) of 5.6, bulk density of 1.2 g cm^–3^, cation exchange capacity of 20.5 cmol (+) kg^–1^, soil organic carbon (SOC) of 14.3 g kg^–1^, and total N of 1.7 g kg^–1^.

### Fertilization Treatment

The fertilizer application treatments included no fertilizer (NF) as a control, chemical fertilizer only (CF), chemical fertilizer plus rice straw return (CFS), and chemical fertilizer plus pig manure (CFM), as described elsewhere (Pan et al., 2009). As per the conventional rates by local farmers, chemical fertilizers were applied at in total 427.5 kg N ha^–1^, 45 kg P_2_O_5_ ha^–1^ and 84 kg K ha^–1^ every year, with 60% allocated for rice and the other 40% for rape. Meanwhile, rice straw was applied at 4.5 t ha^–1^ for CFS and swine manure at 16.8 t ha^–1^ (fresh weight) for CFM, every year. Swine manure was collected from a household pig farm, and fresh rice straw after harvest was chopped before use. Both chopped rice straw and swine manure were spread onto topsoil and merged into a depth of 0–20 cm with manual operation before rape seedling transplantation. Triplicated subplots (each 66.7 m^2^ in area) for each treatment were arranged in a completely randomized block design. All the farming practices including irrigation and plant protection were consistent across the treatments. The basic properties of topsoil under the treatments are listed in Table 1.

**TABLE 1:** Basic properties of bulk soil samples under different treatments.

Treatment	pH (H_2_O)	SOC (g/kg)	TN (g/kg)	MWD (μm)	MBC (mg/kg)	MBN (mg/kg)
NF	5.98 ± 0.08 a	17.51 ± 0.77 b	1.67 ± 0.08 b	534.81 ± 21.57 c	487.95 ± 29.81 b	29.90 ± 2.69 a
CF	5.60 ± 0.05 b	20.67 ± 0.67 a	2.01 ± 0.02 a	577.51 ± 14.22 b	466.22 ± 17.42 b	27.36 ± 3.03 a
CFM	5.48 ± 0.05 b	20.79 ± 0.70 a	1.93 ± 0.05 a	621.48 ± 13.62 a	478.77 ± 30.35 b	27.78 ± 5.17 a
CFS	5.22 ± 0.03 c	21.31 ± 0.50 a	2.00 ± 0.01 a	592.33 ± 9.39 ab	560.84 ± 23.99 a	33.00 ± 4.43 a

*Values as mean ± SD (n = 3). Different letters in a single column indicate significant (p < 0.05) difference among treatments. Soil pH is measured with 1:2.5 (m/v) soil-to-water ratio. SOC, soil organic carbon; TN, total nitrogen; MWD, mean weight diameter; MBC, microbial biomass carbon; MBN, microbial biomass nitrogen.*

### Soil Sampling

Field moist topsoil (0–15 cm) samples were collected at rice harvest in early November 2013. In each plot, five undisturbed bulk soil cores were randomly taken with a soil core sampler and pooled and homogenized as a composite sample of approximately 1 kg. All samples were sealed in stainless steel cans, stored in an icebox, and shipped to the laboratory within 24 h following sampling. After removal of plant debris, a sample was gently crushed into small clods of about 10 mm in size and further homogenized. Subsequently, the sample was divided into four portions by quadrats. One portion was frozen-dried and then stored at −70°C prior to DNA extraction. Another portion was sieved through a 2-mm mesh and air-dried prior to treatment for basic physicochemical determination. The remnant two portions were pooled and directly stored in a stainless steel jar at 4°C before aggregate-size fractionation and microbial biomass determination for bulk soil.

### Aggregate-Size Fractionation

Size fractionation of soil-stable aggregates was performed with a wet-sieving protocol using a series of nylon sieves, respectively, in sizes of 2,000, 250, and 53 μm in distilled water, based on Stemmer et al. (1998) with modifications after Fansler et al. (2005) and Lobe et al. (2011). In detail, a 300-g equivalent moist bulk sample was gently and evenly put on the top of a nylon sieve of 2-mm mesh size and submerged for 10 min in water at room temperature. The sieves were gently moved up and down by 3 cm to the water table for 250 times. Three fractions of water-stable soil aggregates remaining on each sieve were, respectively, collected as large MacA (L-MacA) in size of >2,000 μm, MacA in size of 250–2,000 μm, and microaggregates (MicA) in size of 250–53 μm. Subsequently, the soil suspension passed through the 53-μm sieve was centrifuged at 600 revolutions per minute [rpm] (86 relative centrifugal force [rcf] × *g*) for 4 min in 500-mL plastic centrifuge bottles to collect fine microaggregates (F-MicA) in size of 53–2 μm. The subsequent suspension was again centrifuged at 4,200 rpm (4,219 rcf × *g*) for 36 min in 50-mL centrifuge tubes to obtain the fine clay (F-Clay) particles in size <2 μm. Finally, each obtained size fraction sample was frozen-dried and stored in polyethylene bags at −70°C prior to physicochemical determination and DNA extraction.

### Soil Properties Analysis

Analysis of basic properties of bulk soil and aggregate fraction samples was done following the methods described by Lu (2000). Soil moisture content was measured by oven-drying at 105°C for 12 h. SOC content was determined with wet digestion and dichromate oxidation, whereas total nitrogen (TN) with Kjeldahl method. Soil pH was determined in suspension with the soil-to-water ratio of 1:2.5 (m/v) using a Mettler Toledo pH meter (SevenEasy S20K, Mettler Toledo Inc, 2008). Microbial biomass carbon (MBC) and nitrogen (MBN) contents were determined using the fumigation–extraction protocol (Brookes et al., 1985). Differences in carbon and nitrogen contents between fumigated and unfumigated samples were calculated and converted to contents of MBC and MBN, respectively, with a coefficient of 0.38 and 0.45.

### DNA Extraction and Quantification of Bacteria and Fungi Population

Total DNA of soil aggregate-size fraction samples was extracted from 0.25 g equivalent dry soil using the PowerSoil^®^ DNA Isolation Kit (MO BIO Laboratories Inc., Carlsbad, CA, United States). The extracted DNA was qualified by gel electrophoresis and quantified using a NanoDrop 2000 spectrophotometer (Thermo Scientific, MA, United States) and then stored at −70°C prior to molecular assays. Quantification of bacterial 16S rDNA and fungal 18S rDNA was performed on a 7500 real-time polymerase chain reaction (PCR) system (Applied Biosystems, United States) using SYBR^®^ Premix Ex Taq™ II kit (TAKARA). Specifically, universal primer pairs of 338F (ACTCCTACGGGAGGCAGCAG)/518R(ATTACCGCGGCTG CTGG) (Fierer et al., 2005) and NS1F (GTAGTCATATGCT TGTCTC)/FungR (ATTCCCCGTTACCCGTTG) (May et al., 2001) were used to target bacterial 16S and fungal 18S rRNA genes, respectively. The quantitative PCR conditions included an initial denaturation at 95°C for 5 min, followed by denaturation at 95°C for 45 s for 35 cycles, and annealing at 56°C for bacteria and 57°C for fungi for 30 s, respectively. Standard curves were generated with triplicated 10-fold dilution series of plasmids with the corresponding target genes. The *R*^2^ of each standard curve exceeded 0.99, and each PCR efficiency was between 90 and 110%.

### Amplicon Sequencing of Bacterial and Fungal House-Keeping Genes

The amplicon sequencing library was prepared by targeting the V4–V5 hypervariable region of bacterial 16S rRNA gene and/or the ITS1 region of fungal ITS. Primer pairs of 515F (GTGCCAGCMGCCGCGG)/907R(CCGTCAATTCMTTTRAG TTT) were chosen to flank the bacterial target region, whereas the fungal ITS region was captured by primer pairs of ITS1F (CTTGGTCATTTAGAGGAAGTAA)/ITS2(GCTGCGTTCTTC ATCGATGC). A 20-μL mixture was prepared for each PCR reaction, including 1× reaction buffer (TAKARA), 2 mM Mg^2+^, 0.2 mM dNTP, 0.1 μM of each primer, 1 U HotStar Taq polymerase (TAKARA), and 2 μL template DNA, followed by 95°C for 2 min; 35 cycles of 94°C for 20 s, 55°C for 40 s, 72°C for 1 min, and 72°C for 2 min. The PCR products were then subject to adapter annealing where a unique 6-bp barcode was added by a second PCR reaction. Each reaction contained 1 × reaction buffer (NEB Q5™), 0.3 mM dNTP, 0.25 μM of F primer, 0.25 μM of index primer, 1 U Q5™ DNA polymerase, and 1 μL diluted template. The PCR conditions included an initial denaturation at 98°C for 30 s followed by 11 cycles of (i) denaturation (98°C for 10 s), and (ii) annealing (65°C for 30 s), and extension (72°C for 30 s). The PCR ended with a final extension at 72°C for 5 min. Paired-end sequence data were assembled by *FLASH* (Magoc and Salzberg, 2011) after masking low-quality end bases (<Q15) with the *FASTX-Toolkit*^1^. Barcodes and primers were trimmed with *cutadapt* (Martin, 2011). Sequences shorter than 200 bp and with a quality score <20 were discarded, and chimeras and singletons were filtered with the *UPARSE* algorithm (Edgar, 2013). The remaining reads were then clustered into operational taxonomic units (OTUs) at 97% similarity cutoff via *UPARSE* with default settings. Taxonomy was assigned to bacterial OTUs by RDP^2^ or to fungal OTUs by *UNITE*^3^. Each sample was rarefied before downstream analyses. Shannon (H) and Good’s coverage index were calculated at the OTU level using *Mothur* (Kozich et al., 2013). Sequence data from this study have been deposited to the NCBI Sequence Read Archive (SRA) under the accession numbers of PRJNA591295 and PRJNA591298 for bacterial and fungal communities, respectively.

### Microbial Composition and Community Structure Assessment

Relative abundances were determined by dividing the number of sequences assigned to each category classified at the phylum level (phylum Proteobacteria was classified at class level) by the total number of sequences within each sample. Average proportions less than 1% were affiliated to category others. Non-metric multidimensional scaling (NMDS) (Faith et al., 1987) was generated in the R package vegan (Oksanen et al., 2007) via the *metaMDS* function to compare and visualize the microbial composition across samples. To quantitatively evaluate the impacts of fertilization treatments and aggregate-size fractions on bacterial and fungal community structures, a forward permutational multivariate analysis of variance (PERMANOVA; 999 permutations) (Anderson, 2001) was performed via the *adonis* function in the *vegan* package. Soil chemical properties (SOC, TN, C/N ratio) were fitted onto the NMDS ordinations by the *envfit* function.

### Analysis of Microbial Co-occurrence Network and Community Functional Prediction

Microbial co-occurrence in each size fraction was examined by network analysis. To reduce network complexity, only the OTUs with more than five reads and presented in more than two-thirds samples were kept to generate Spearman rank correlation coefficient matrices using the *WGCNA* package (Langfelder and Horvath, 2012). Corresponding *p* values of the correlation coefficient matrices were adjusted into *q* values by Benjamini–Hochberg’s false discovery rate (FDR) in the *fdrtool* package to minimize false-positive rates. Co-occurrence networks were constructed based on correlation coefficient matrices with *q* < 0.01 or | ρ| > 0.6 (Barberan et al., 2012; Ma et al., 2016) in the *igraph* package (Csardi and Nepusz, 2006) and visualized using Gephi (version 0.9.2) (Bastian et al., 2009). Unconnected nodes along with loops that indicate OTUs correlated with themselves were, respectively, removed using the *delete.vertices* and *simplify* functions. The topological features used to describe network properties were assessed by following parameters using the corresponding functions in *igraph* (Newman, 2003, 2006). Specifically, the number of nodes denoted the number of OTUs with at least one correlation, and the number of edges denoted the number of connections among the nodes obtained, whereas the average degree indicated the average number of connections through each node. Average path length denoted the average network distance among the nodes, whereas the diameter denoted the longest distance among the nodes. Furthermore, clustering coefficient was the degree to which nodes in a network tended to cluster together. Finally, a parameter of modularity was used to represent the network structure indicating the strength of division of a network into modules. At last, the resulting networks were exported in GRAPHML format for further optimization and visualization in Gephi version 0.9.2 (Bastian et al., 2009). The network graphs were eventually generated using the Fruchterman-Reingold layout by Gephi and resized according to the parameter of diameter by Adobe Illustrator.

Soil bacterial functional profile was predicted using PICRUSt (Phylogenetic Investigation of Communities by Reconstruction of Unobserved States) from the 16S rRNA marker gene sequence data (Langille et al., 2013). Such work was based on the databases of Cluster of Orthologous Groups of proteins (COGs) (Tatusov et al., 2000) and Kyoto Encyclopedia of Genes and Genomes (KEGG) (Kanehisa and Goto, 2000) to infer gene content and then to predict the bacterial metagenome functional content annotated into COG and KEGG pathway categories, respectively, whereas an open annotation tool, FUNGuild (Fungi functional guild), was used to taxonomically parse fungal community OTUs into ecological functional guild with the python script Guilds v1.1, which run against the FUNGuild database (Nguyen et al., 2016). The annotations with confidence ranking of “Highly Probable” and “Probable” were used for the prediction of the community functional guilds. Differences of the functional groups between each size fraction of aggregate samples and all the other samples were compared with two-sided Welch *t* test with 0.95 confidence interval (CI) and Benjamini–Hochberg FDR for multiple tests and then plotted with features at *q* < 0.05 by the software STAMP 2.1.3 (Parks et al., 2014; Douglas et al., 2018).

### Data Processing and Statistics

Data processing was performed with Microsoft Excel 2016. All data of a sample were expressed as mean plus/minus a standard deviation (*n* = 3). Statistical analyses were conducted by GraphPad Prism 7 and SPSS 24. Figures were plotted with Excel 2016 and R 3.5.0. Differences among different fertilization treatments and aggregate-size fractions were analyzed with one-way or two-way analysis of variance (ANOVA). Subsequently, multiple comparisons were conducted using Tukey test to determine the significance defined at *p* < 0.05.

## Results

### Basic Properties of Aggregate-Size Fractions

Compared with NF, contents of SOC and TN were all significantly increased, whereas pH decreased, although no change was observed in C/N ratio under the long-term application of fertilizers (Table 1). Meanwhile, MBC was significantly increased under CFS but MBN was unchanged with the treatments. The separation protocol yielded an overall recovery in the range of 94.23–99.45%. Across the samples, water-stable aggregates were dominated by F-MicA (32.3–38.2%) and MacA (23.0–31.5%), followed by MicA (19.5–23.1%) and L-MacA (9.1–12.0%), with the least contribution by F-Clay (4.9–7.5%) (Table 2). Except for the F-Clay fraction, the mass proportion of an aggregate-size fraction varied greatly across the treatments. With fertilization compared with no fertilization, the mass proportion of MacA was significantly increased by 19.2–37.3%, but that of F-MicA was reduced by 14.0–15.5%, and that of the other fractions was insignificantly unchanged. Consequently, the mean weight diameter (MWD) of water-stable soil aggregates (Table 1) was increased from 534.8 μm under NF to 577.5–621.5 μm by 8.0–16.2% under fertilizer treatments. Thus, the mass proportion of soil aggregate fractions varied greatly with size but significantly affected, mainly for MacA and F-MicA fractions, with fertilization practice.

**TABLE 2:** Mass proportion and basic properties (mean ± SD, *n* = 3) of aggregate-size fractions under different fertilization treatments.

	Treatment	>2,000 μm	250–2,000 μm	53–250 μm	2–53 μm	<2 μm
Mass proportion (%)	NF	11.81 ± 1.36 ^cA^	22.97 ± 1.03 ^bB^	19.52 ± 2.52 ^bA^	38.22 ± 3.13 ^aA^	7.47 ± 0.40 ^cA^
	CF	9.07 ± 1.89 ^cA^	31.54 ± 2.49 ^aA^	21.27 ± 1.91 ^bA^	32.87 ± 2.70 ^aB^	5.25 ± 1.82 ^cA^
	CFM	11.92 ± 1.29 ^cA^	30.70 ± 2.01 ^aA^	18.98 ± 3.46 ^bA^	32.29 ± 2.97 ^aB^	6.11 ± 0.81 ^dA^
	CFS	12.01 ± 0.83 ^cA^	27.39 ± 1.74 ^bA^	23.10 ± 1.40 ^bA^	32.66 ± 1.53 ^aB^	4.85 ± 0.92 ^dA^
SOC (g/kg)	NF	16.41 ± 0.65 ^bC^	20.91 ± 1.23 ^aB^	18.87 ± 2.15 ^abA^	12.24 ± 0.37 ^cA^	18.52 ± 0.30 ^abA^
	CF	21.83 ± 1.55 ^abA^	23.29 ± 1.57 ^aAB^	20.70 ± 1.30 ^abA^	14.58 ± 0.91 ^cA^	19.67 ± 1.47 ^bA^
	CFM	20.78 ± 1.15 ^aAB^	21.30 ± 1.69 ^aAB^	19.80 ± 0.96 ^aA^	13.88 ± 1.01 ^bA^	20.10 ± 1.13 ^aA^
	CFS	18.61 ± 0.67 ^bBC^	23.66 ± 1.00 ^aA^	21.16 ± 1.09 ^abA^	14.52 ± 0.66 ^cA^	20.18 ± 0.80 ^bA^
TN (g/kg)	NF	1.64 ± 0.05 ^bB^	1.99 ± 0.12 ^aA^	1.61 ± 0.19 ^bB^	1.40 ± 0.03 ^bA^	2.12 ± 0.07 ^aA^
	CF	2.10 ± 0.24 ^aA^	2.21 ± 0.22 ^aA^	1.99 ± 0.29 ^aA^	1.62 ± 0.04 ^bA^	2.20 ± 0.25 ^aA^
	CFM	2.02 ± 0.17 ^abA^	1.98 ± 0.18 ^abA^	1.73 ± 0.16 ^bcAB^	1.57 ± 0.09 ^cA^	2.24 ± 0.10 ^aA^
	CFS	1.79 ± 0.03 ^cAB^	2.16 ± 0.09 ^abA^	1.82 ± 0.13 ^bcAB^	1.62 ± 0.04 ^cA^	2.19 ± 0.10 *^aA^*

*Different lowercase or uppercase letters in a single line or column denote a significant (p < 0.05) difference among aggregate classes or treatments.*

The contents of SOC and total N of soil aggregates varied moderately (CV 12–20%) across the size fractions. Generally, the SOC content peaked in MacA (20.91–23.66 g kg^–1^) but depleted in F-MicA (12.24–14.58 g kg^–1^) with no significant difference among the fractions of MicA, L-MacA and F-Clay. The contents of TN followed a similar trend, although enriched in the F-Clay fraction. However, the change in these contents with the treatments was insignificant across the size fractions of MicA, F-MicA and F-Clay despite marked (CV > 10%) changes found only in L-MacA. Compared with NF, the SOC content of MacA was increased by 13.2% under CFS, and that of L-MacA by 33.0% under CF and by 26.6% under CFM. Unlike the SOC content, the total N (TN) content of L-MacA was increased by 9.1% under CFS and by 23.2–28.0% under CFM and CF, whereas that of MicA was increased by 22.3% under CF only. Consequently, the C/N ratio showed a slight (CV ∼10%) variation across the size fractions but a weak variation with fertilization treatments. Generally, the C/N ratio was highest (10.4–11.7) in MicA, followed by MacA and L-MacA (10.0–10.9), and lowest in the F-MicA and F-Clay fractions (8.7–9.2). Under CF, particularly, the C/N ratio was significantly lower in MicA compared with other fertilizer applications (Table 2). Overall, the aggregate size factor caused a greater contribution (by 62.6–93.8%) than the treatment factor with a slight contribution (by 0.0–12.0%) to the overall variation of aggregate properties, as shown in Supplementary Table 1.

### Microbial Population Abundance and Community Structure

The population sizes of both bacteria and fungi, quantified by 16S and 18S rRNA gene copy numbers, respectively (Table 3), demonstrated a broad range of variation across aggregate-size fractions (CV 51–98%), decreasing generally with the sizes, and among fertilizer treatments (CV 3–123%) (Table 3). In a range of ≤0.5 × 10^9^ to 5.10 × 10^9^ copies g^–1^ dw and of ≤0.2 × 10^7^ to 10.2 × 10^7^ copies g^–1^ dw, the abundance of both bacterial 16S and fungal 18S rRNA gene was lowest in F-Clay but highest in MacA (for bacteria) or in L-MacA (for fungi). Despite no change was found among treatments in L-MacA, MacA, and MicA, both bacterial 16S and fungal 18S rRNA gene copy numbers were highly increased by fertilizer application in F-MicA (2- to 3-fold for bacteria, 4- to 5-fold for fungi), while increased under CF but decreased under CFM and CFS by folds in F-Clay (Table 3). In addition, the B/F ratio, ranging from 45.97 to 611.23, was lowest in L-MacA or MacA and increased with the decreasing size of the aggregate fractions. Meanwhile, the ratio was seen to decrease under fertilizer application only in F-MicA fraction, without treatment induced changes in those >53-μm fractions. Based on the two-way ANOVA, the aggregate size factor exerted a greater effect (*p* < 0.0001) than the treatment factor (*p* < 0.01), on the bacterial abundance, fungal abundance, and B/F ratio, which were, respectively, contributed by 83.3, 73.5, and 64.1% by size factor, whereas 4.0, 6.5, and 7.4% by treatment factor to the total variation (Supplementary Table 2).

**TABLE 3:** Bacterial 16S (10^9^ copies g^–1^ dw) and fungal 18S (10^7^ copies g^–1^ dw) gene abundances (mean ± SD, *n* = 3) of aggregate-size fractions under different treatments.

	Treatment	>2,000 μm	250–2,000 μm	53–250 μm	2–53 μm	<2 μm
Bacterial	NF	4.29 ± 0.66 ^aA^	3.93 ± 0.39 ^aA^	3.14 ± 0.68 ^aA^	0.98 ± 0.08 ^bB^	0.38 ± 0.08 ^bB^
	CF	4.60 ± 0.78 ^aA^	4.76 ± 1.10 ^aA^	3.92 ± 0.98 ^aA^	2.96 ± 0.73 ^aA^	0.54 ± 0.04 ^bA^
	CFM	4.31 ± 0.69 ^abA^	5.10 ± 0.72 ^aA^	4.65 ± 1.00 ^aA^	2.86 ± 0.29 ^bA^	0.01 ± 0.00 ^cC^
	CFS	4.48 ± 0.21 ^aA^	4.54 ± 0.45 ^aA^	4.14 ± 0.31 ^abA^	3.54 ± 0.29 ^bA^	0.05 ± 0.00 ^cC^
Fungal	NF	8.21 ± 1.01 ^aA^	5.13 ± 0.88 ^bA^	3.36 ± 1.27 ^bA^	0.47 ± 0.11 ^cB^	0.11 ± 0.05 ^cB^
	CF	10.20 ± 2.68 ^aA^	8.53 ± 3.38 ^abA^	7.20 ± 3.58 ^abA^	2.49 ± 0.60 ^bcA^	0.24 ± 0.03 ^cA^
	CFM	9.15 ± 3.12 ^aA^	8.81 ± 3.26 ^aA^	7.10 ± 2.72 ^aA^	2.81 ± 0.29 ^abA^	0.00 ± 0.00 ^bC^
	CFS	8.72 ± 1.51 ^aA^	4.82 ± 0.54 ^bA^	4.55 ± 1.40 ^bA^	2.46 ± 0.69 ^bcA^	0.01 ± 0.00 ^cC^

*Different lowercase and uppercase letters in a single line and column represent a significant (p < 0.05) difference among aggregate classes and treatments, respectively.*

**FIGURE 1:**
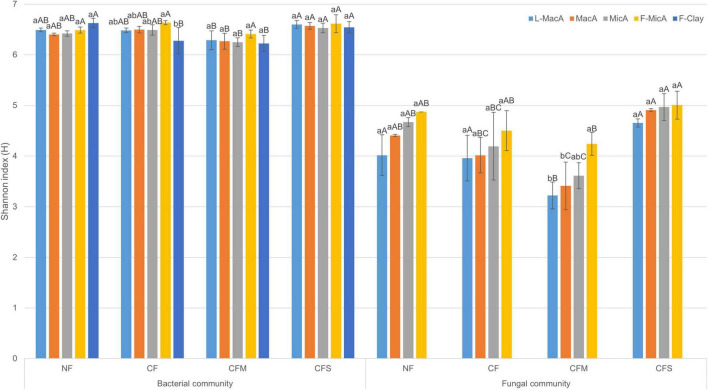
Shannon index (H) (mean ± SD, *n* = 3) of bacterial and fungal communities in aggregate-size fractions under treatments. Different lowercase and uppercase letters represent significant (*p* < 0.05) difference among aggregate-size fractions and treatments, respectively. NF, no fertilizer applied; CF, compound inorganic fertilizer; CFM, inorganic fertilizer combined with swine manure; CFS, inorganic fertilizer combined with residue return. L-MacA, >2,000 μm; MacA, 2,000–250 μm; MicA, 250–53 μm; F-MicA, 53–2 μm; F-Clay, <2 μm.

Moreover, the Shannon diversity indices were generally greater for bacteria population than fungi for a given aggregate fraction (Figure 1). Regardless of fertilization treatment, the Shannon indices of fungi of an aggregate fraction showed an insignificantly increasing trend with decreasing size, whereas that of bacterial was similar across the size fractions. In addition, the diversity index for both bacteria and fungi was lower under CFM compared with the other fertilization treatments, across the size fractions (Figure 1). Overall, microbial community diversity among aggregate fractions was rather impacted by fertilization than by aggregates size, and there was no significant interaction between size and treatment (Supplementary Table 3).

**FIGURE 2:**
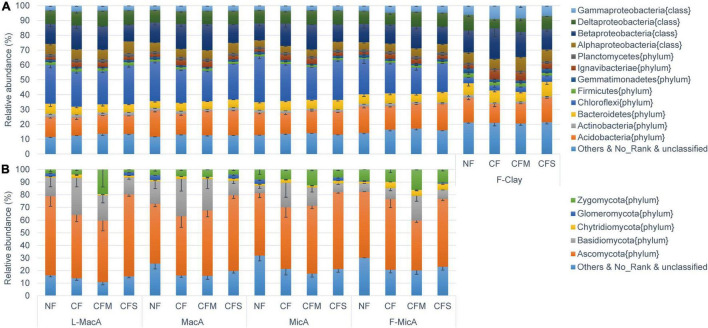
Average relative abundance distribution of V4–V5 16S rDNA sequence classified at phylum level (class for Proteobacteria) for bacteria **(A)** and ITS1-ITS2 sequence classified at phylum level for fungi **(B)**. Error bars represent the standard errors. Average proportions of showed bacterial phyla (class for Proteobacteria) and fungal phyla all exceed 1%. NF, no fertilizer applied; CF, compound inorganic fertilizer; CFM, inorganic fertilizer combined with swine manure; CFS, inorganic fertilizer combined with residue return. L-MacA, >2,000 μm; MacA, 2,000–250 μm; MicA, 250–53 μm; F-MicA, 53–2 μm; F-Clay, < 2 μm.

The bacterial population detected in soil aggregates across size fractions was dominated by Chloroflexi (3.5–30.8%), Betaproteobacteria (11.2–20.6%), Acidobacteria (12.0–17.9%), and Deltaproteobacteria (6.3–10.6%) (Figure 2A). For bacteria assembly, the population of Chloroflexi was smaller, but that of gamma-proteobacteria was larger in the F-Clay fraction than in the MicA and MacA fractions. For fertilizer treatment, the population of Chloroflexi was larger, and that of Betaproteobacteria was smaller under the NF and CFS treatments than under the CF and CFM treatments (Figure 2A). The fungal community was predominated by Ascomycota (39.8–64.9%) and Basidiomycota (35.6–59.7%) across the aggregate samples (Figure 2B). Hereby, the relative abundance of fungal communities in a fraction was much variable for the larger standard deviation compared with bacterial communities. Specifically, Basidiomycota in all the aggregate fractions, except for the F-Clay fraction, showed a significantly higher relative abundance under CF than under the other treatments. Differently, the abundance of *Ascomycota* was significantly lower in L-MacA under CF and CFM, and in F-MicA under CFM, respectively, compared with under NF or CFS. However, the relative abundance of Zygomycota, a minor fungal phylum, appeared several folds higher in L-MacA and F-MicA under CFM over the other fertilizer treatments (Figure 2B). Besides, there was a generally lower relative abundance of unspecified fungal phyla under fertilizer applications compared with NF.

**FIGURE 3:**
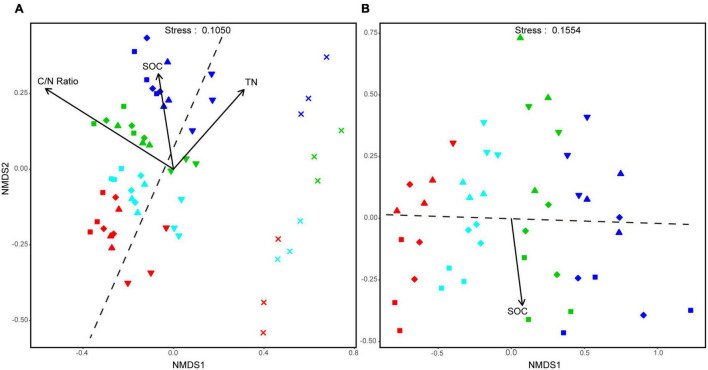
Non-metric multidimensional scaling (NMDS) plots of all the detected samples at OTU level of the aggregate-size fractions, respectively, for the bacterial **(A)** and fungal community **(B)**. Symbols denote size fractions as follows: ■, >2,000 μm; ◆, 2,000–250 μm; ▲, 250–53 μm; ▼, 53–2 μm; ×, <2 μm. Colors denote treatments as follows: red, NF; green, CF; blue, CFM; black, CFS. Dotted lines, respectively, indicate the boundary between MicA and F-MicA in A while between MacA and MicA in panel **B**.

Based on NMDS, the bacterial community assembly of aggregate-size fractions was clearly influenced by both soil aggregate size and fertilization treatment. Especially, the bacterial communities in F-MicA and F-Clay were distinct from larger-sized aggregate fractions hardly altered with the treatments (Figure 3A). Moreover, the bacterial community structures of the L-MacA, MacA, and MicA fractions were similar in NMDS1, whereas F-MicA and/or F-Clay housed bacteria communities whose structures were similar to each other than to those in larger aggregates (Figure 3A). However, the bacterial community structures were altered with fertilization by NMDS2. Thereby the similarity of bacterial community structure with the treatments was in an order of NF, CFS, CF, and CFM. In contrast, size factor showed minor influence on fungal community structures of soil aggregates, relative to fertilization treatment (Figure 3B). The fungal community structures were altered with fertilization by NMDS1, whereas the treatment effect was greater under CF and CFM than under CFS, compared with NF. Meanwhile, the fungal community structures were altered more or less with the size factor by NMSD2, showing the MicA and F-MicA fractions were relatively separated from the L-MacA and MacA fractions (Figure 3B). Based on PERMANOVA, the bacterial community structure was significantly attributed to C/N ratio (*p* < 0.001), SOC (*p* < 0.05), and TN (*p* < 0.05), whereas the fungal community similarity was statistically correlated to SOC (*p* < 0.01) (Figure 3).

### Microbial Co-occurrence Networks Among Aggregate-Size Fractions

**FIGURE 4:**
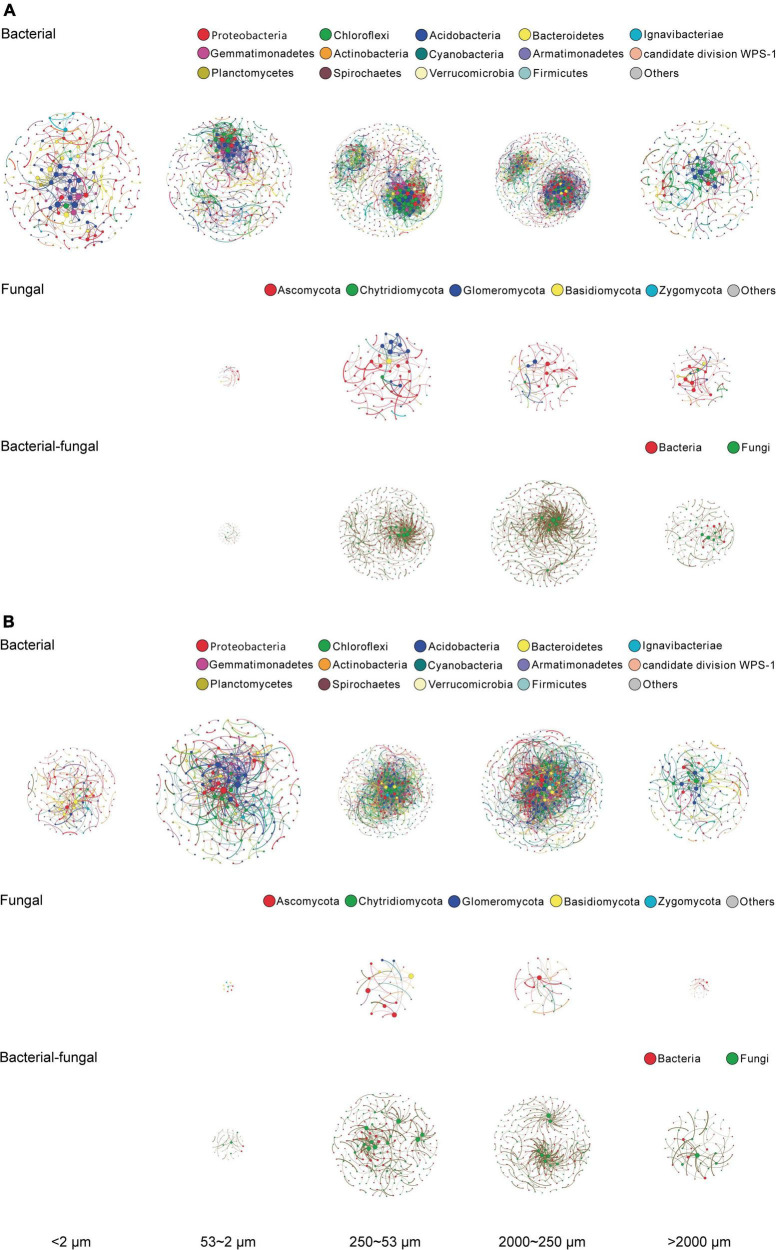
Positive **(A)** and negative **(B)** co-occurrence networks of bacterial, fungal, and bacterial–fungal consortia of the size fractions of aggregates based on Spearman rank correlation coefficient matrices. Colored nodes indicate OTUs represented by phyla. An edge stands for a strong positive (Spearman ρ > 0.6) and significant (*p* < 0.01) correlation between the two nodes. For each network, the size of each node is proportional to the number of edges (degree). The size of a network for a size fraction is proportional to the size of the corresponding networking scope. The thickness of each edge is proportional to the correlation coefficient.

Both SOC and microbial abundance of soil aggregate fractions were shaped predominantly by size (see *Basic properties of a*ggregate-size fractions and *Microbial population abundance and community structure*), thereby the microbial OTUs of a single fraction of individual samples under different treatments were pooled as a single sample for analysis of co-occurrence networks. After filtering all the OTU data, in total 639–763 bacterial OTUs and 229–305 fungal OTUs were obtained, respectively, for network construction of bacterial and fungal community for the aggregate-size fractions, based on the significant (*p* < 0.01) and strong (ρ > 0.6 or ρ < −0.6) correlations as per Barberan et al. (2012). The bacterial co-occurrence networks were dominated by OTUs affiliated with the phyla of Proteobacteria, Chloroflexi, Acidobacteria, Bacteroidetes, and Ignavibacteriae (Figure 4), whereas the fungal networks were centered around Ascomycota, Chytridiomycota, Glomeromycota, Basidiomycota, and Zygomycota (Figure 4). The bacterial–fungal co-occurrence networks were attributed to 52.9–69.2% of bacterial and 30.8–47.1% of fungal communities across the size fractions (Figure 4). Generally, the microbial co-occurrence networks exhibited disparate patterns among size fractions, and both the positive and negative co-occurrence networks were much more complicated for the bacterial communities than the fungal communities (Figure 4). Both positive and negative correlations in bacterial, fungal, and bacterial–fungal networks were observed in all the aggregate fractions other than F-Clay with fungal and bacterial–fungal networks and significantly in MicA, MacA and L-MacA. As indicated by the edge thickness, the positive networking was relatively stronger than negative networking across the size fractions, particularly in MacA and L-MacA. The numbers of nodes, edges, and the average degree of the networks were all higher in MacA and MicA, compared with other fractions (Supplementary Tables 6, 7). However, more intricate networks led to lower modularity in MacA and MicA with a similar number of clusters to the other fractions. For MacA and MicA compared with the other fractions, the average path length of bacterial network was lower, whereas that of fungal and bacterial–fungal networks was higher. The opposite trend of density and clustering coefficient across size fractions showed more compact bacterial networks but looser fungal and bacterial–fungal networks in MacA and MicA compared with the other size fractions. Overall, microbial communities exerted higher networking performance in MacA than MicA and for bacterial than fungal and bacterial–fungal networks.

### Microbial Community Functional Profiles Among Aggregate-Size Fractions

Summarized from the statistical results in Supplementary Figures 1, 2, the difference in the functional profiles of COGs and KEGG metabolic pathways for bacterial communities among the aggregate-size fractions is shown in Tables 4, 5, respectively. L-MacA, MacA, and MicA all possessed more bacterial populations with functional genes encoding enzymes involving transport and metabolism of inorganic ion and various types of carbohydrate, transport proteins, and RNA processing and modification, but were less abundant of genes related to bacterial chemotaxis, cell motility, flagellar assembly, protein biosynthesis, signal transduction, intracellular trafficking, secretion and vesicular transport, DNA repair and recombination, and so on. However, F-MicA possessed a higher abundance of functional genes encoding enzymes involving defense mechanisms and metabolism of certain sugars and amino acids, while possessed a lower abundance of genes with functions of inorganic ion transport and metabolism and cell chemotaxis, and so on. Bacterial populations with functional genes associated with bacterial chemotaxis, cell motility, flagellar assembly, and protein biogenesis were predominantly detected in F-Clay. Meanwhile, this fraction possessed a lower abundance of genes functioning in transport and metabolism of carbohydrate and amino acid, defense mechanisms, RNA processing and modification, transport proteins, and so on.

**TABLE 4:** Summary of the PICRUSt results of 16S rDNA at COG level.

	More	Less
F-Clay	[N] Cell motility; [J] Translation, ribosomal structure and biogenesis; [T] Signal transduction mechanisms; [U] Intracellular trafficking, secretion, and vesicular transport; [H] Coenzyme transport and metabolism; [I] Lipid transport and metabolism; [L] Replication, recombination and repair; [D] Cell cycle control, cell division, chromosome partitioning; [W] Extracellular structures	[G] Carbohydrate transport and metabolism; [R] General function prediction only; [E] Amino acid transport and metabolism; [V] Defense mechanisms; [A] RNA processing and modification
F-MicA	[V] Defense mechanisms	[P] Inorganic ion transport and metabolism
MicA	[G] Carbohydrate transport and metabolism; [V] Defense mechanisms; [F] Nucleotide transport and metabolism; [A] RNA processing and modification	[N] Cell motility; [U] Intracellular trafficking, secretion, and vesicular transport; [T] Signal transduction mechanisms; [I] Lipid transport and metabolism; [H] Coenzyme transport and metabolism; [W] Extracellular structures
MacA	[G] Carbohydrate transport and metabolism; [A] RNA processing and modification	[J] Translation, ribosomal structure and biogenesis; [H] Coenzyme transport and metabolism; [F] Nucleotide transport and metabolism; [W] Extracellular structures
L-MacA	[P] Inorganic ion transport and metabolism; [A] RNA processing and modification; [B] Chromatin structure and dynamics	[M] Cell wall/membrane/envelope biogenesis; [U] Intracellular trafficking, secretion, and vesicular transport; [V] Defense mechanisms; [F] Nucleotide transport and metabolism

*Summary of Supplementary Figure 1. Content in each lattice displays the COG pathways of each sized fraction significantly more or less than all the other aggregate samples. Analyses were conducted in the software STAMP with statistical method of two-sided Welch t test with 0.95 CI and Benjamini–Hochberg FDR for multiple tests. Corrected p (q) < 0.05.*

**TABLE 5:** Summary of the PICRUSt results of 16S rDNA at KEGG level.

	More	Less
F-Clay	Bacterial motility proteins; secretion system; two-component system; flagellar assembly; bacterial chemotaxis; chromosome; ribosome biogenesis; ribosome; lipopolysaccharide biosynthesis proteins; pyrimidine metabolism; lipopolysaccharide biosynthesis; bacterial secretion system; purine metabolism; carbon fixation pathways in prokaryotes; nitrogen metabolism; DNA replication proteins; butanoate metabolism; peptidoglycan biosynthesis; riboflavin metabolism; DNA repair and recombination proteins	Transporters; ABC transporters; galactose metabolism; starch and sucrose metabolism; amino sugar and nucleotide sugar metabolism; pentose and glucuronate interconversions; glycerolipid metabolism; methane metabolism; glycolysis/gluconeogenesis; pentose phosphate pathway; fructose and mannose metabolism; sphingolipid metabolism; transcription factors; peptidases; cysteine and methionine metabolism; glycosphingolipid biosynthesis–globo series; other glycan degradation
F-MicA	Amino sugar and nucleotide sugar metabolism; peptidases; transcription machinery; oxidative phosphorylation; galactose metabolism; alanine, aspartate and glutamate metabolism; pentose phosphate pathway; cysteine and methionine metabolism; other glycan degradation; glycine, serine and threonine metabolism; sphingolipid metabolism; histidine metabolism; tyrosine metabolism; phenylalanine, tyrosine and tryptophan biosynthesis; aminoacyl-tRNA biosynthesis	Bacterial chemotaxis; ubiquinone and other terpenoid-quinone biosynthesis; bisphenol degradation; phosphotransferase system (PTS); drug metabolism - cytochrome p450; meiosis—yeast; cell cycle—caulobacter; atrazine degradation
MicA	Transporters; amino sugar and nucleotide sugar metabolism; galactose metabolism; starch and sucrose metabolism; pentose and glucuronate interconversions; glycerolipid metabolism; glycolysis/gluconeogenesis; peptidases; pentose phosphate pathway; fructose and mannose metabolism; sphingolipid metabolism; other glycan degradation; oxidative phosphorylation	Bacterial motility proteins; two-component system; bacterial chemotaxis; butanoate metabolism; limonene and pinene degradation; propanoate metabolism; geraniol degradation; glutathione metabolism; benzoate degradation; lysine degradation; beta-alanine metabolism; glyoxylate and dicarboxylate metabolism; aminobenzoate degradation
MacA	Starch and sucrose metabolism; galactose metabolism; pentose and glucuronate interconversions; glycerolipid metabolism; pentose phosphate pathway; pantothenate and CoA biosynthesis; sphingolipid metabolism; phenylpropanoid biosynthesis; cysteine and methionine metabolism; carotenoid biosynthesis; glycosphingolipid biosynthesis—globo series; sulfur metabolism; penicillin and cephalosporin biosynthesis; phenylalanine metabolism	Carbon fixation pathways in prokaryotes; ribosome; pyruvate metabolism; pyrimidine metabolism; porphyrin and chlorophyll metabolism; citrate cycle (TCA cycle); nitrotoluene degradation; biotin metabolism; pantothenate and CoA biosynthesis; terpenoid backbone biosynthesis; translation factors; proteasome; nicotinate and nicotinamide metabolism; fatty acid biosynthesis
L-MacA	Transporters; ABC transporters; methane metabolism; glycerolipid metabolism; ubiquinone and other terpenoid-quinone biosynthesis; bisphenol degradation; polycyclic aromatic hydrocarbon degradation	Ribosome; flagellar assembly; DNA repair and recombination proteins; Pyrimidine metabolism; lipopolysaccharide biosynthesis proteins; transcription machinery; lipopolysaccharide biosynthesis; oxidative phosphorylation; peptidases; amino sugar and nucleotide sugar metabolism; purine metabolism; alanine, aspartate and glutamate metabolism; riboflavin metabolism; aminoacyl-tRNA biosynthesis; peptidoglycan biosynthesis; phenylalanine, tyrosine and tryptophan biosynthesis

*Summary of Supplementary Figure 2. Content in each lattice displays the KEGG pathways of each sized fraction significantly more or less than all the other aggregate samples. Analyses were conducted in the software STAMP with statistical method of two-sided Welch t test with 0.95 CI and Benjamini–Hochberg FDR for multiple tests. Corrected p (q) < 0.05.*

For fungal communities across aggregate-size fractions, F-MicA contained more functional fungal guilds associated with fungal parasite, litter saprotroph, animal pathogen, endophyte, and epiphyte but less of plant pathogen, arbuscular mycorrhizal, dung saprotroph, and wood saprotroph, whereas larger MacA tended to contain less fungal functional guilds of fungal parasite and litter saprotroph (Table 6 and Supplementary Figure 3).

**TABLE 6:** Summary of the FUNGuild results of ITS1 among size fractions.

Aggregate	More	Less
53–2 μm	Fungal parasite–litter saprotroph; animal pathogen–endophyte–epiphyte–undefined saprotroph	Arbuscular mycorrhizal; plant pathogen–wood saprotroph; dung saprotroph
250–53 μm	n.s	n.s
2,000–250 μm	n.s	n.s
>2,000 μm	n.s	Fungal parasite, litter saprotroph, plant pathogen

*Summary of Supplementary Figure 3. Content in each lattice displays the fungal functional guilds of each sized fraction significantly more or less than all the other aggregate samples. Analyses were conducted in the software STAMP with statistical method of two-sided Welch t test with 0.95 CI and Benjamini–Hochberg FDR for multiple tests. Corrected p (q) < 0.05. Fungal functional guilds occurred at different growth stages during life cycle were linked by symbol “–.”*

## Discussion

### Microbial Abundance in Relation to Soil Organic Matter in MacA

In this study, variation of carbon and microbial abundance among soil aggregate fractions were shaped predominantly by size, although microbial diversity was less variable across the size fractions of aggregates. This is in general agreement with many previous studies of agricultural soils (Whalen and Chang, 2002; Li et al., 2007; Zhang et al., 2007; Hao et al., 2008; Dorodnikov et al., 2009; Lagomarsino et al., 2012). Unlike dryland soils containing significant amounts of coarse sand-sized MacA (Whalen and Chang, 2002; Dorodnikov et al., 2009), soils in this study derived from clayed sediments and were dominated by silt-sized MicA and fine-sand-sized MacA (Table 2). This is attributed to potential stabilization of MicA with water dispersion under long-term hydroagric tillage (Denef et al., 2001; Zhang et al., 2016; Sun et al., 2017). Different to upland soils, where SOC content was enhanced only in fractions with size >63 μm (Marx et al., 2005), consistently high content of SOC was observed in MacA in this study. Similar patterns have been reported in Chinese rice paddies (Li et al., 2007; Zhang et al., 2015; Liu et al., 2016; Lu et al., 2020). Bacterial and fungal populations were more abundant in MacA and L-MacA, compared with those in F-Clay or F-MicA, which is in agreement with the reported microbial populations of rice soils (Zhang et al., 2007; Liu et al., 2016). Therefore, fungal community can be promoted in larger-sized MacA due to physical protection of relatively younger organic matter derived by plant roots with a high C/N ratio (Six et al., 2000; Lützow et al., 2006; Grandy and Robertson, 2007).

**FIGURE 5:**
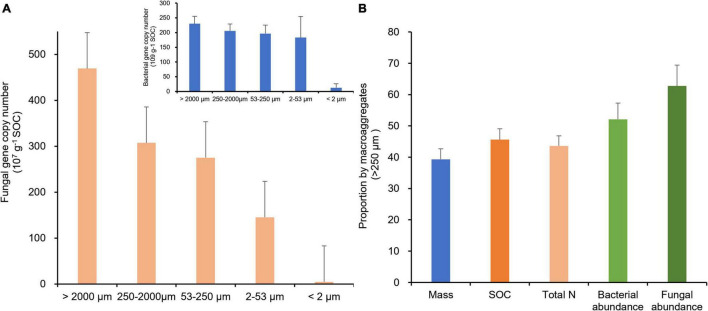
Gene abundance of fungi (**A**, 10^7^ copies g^–1^ SOC) and bacterial (embedded in **A**, 10^9^ copies g^–1^ SOC) in different-size fractions and proportion (%) possessed by macroaggregates > 250 μm **(B)**, of water-stable aggregates of the rice paddy.

Interestingly, when scaled by organic carbon content of aggregate fractions (Table 2), bacterial and fungal abundances were positively correlated with the size of aggregate fractions (Figure 5A), indicating a potential linkage between soil microbial biomass and organic carbon in MacA. With prolonged rice cultivation, wetland converted rice paddy can enhance both the microbial abundance and activity concerning organic carbon accumulation (Jin et al., 2012). In a previous study (Liu et al., 2016), soil microbial abundance and activity were promoted by mainly POM accumulation in MacA, which is triggered via SOC accumulation under rice paddy management over the centuries. Consequently, as argued by Ranjard et al. (2000), up to 90% soil bacteria are associated with MacA in soil. On the other hand, MicA and F-Clay particles have been well known for their enriched mineral-associated organic matter (Cotrufo et al., 2013), which are inaccessible to microbes, particularly fungi. Considering the mass distribution, SOC, and microbial abundance by size fractions of soil aggregates, the relative proportion by MacA of > 250 μm in size was significantly higher than its mass proportion for the abiotic property of SOC and total N but greatly higher for the biotic property of bacterial and fungal abundance (Figure 5B).

Thus, we see the existence of biotic “hotspot” for MacA at the microscale of soil matrix, first proposed by Bundt et al. (2001) for enriched younger OM in pores within MacA, where active biogeochemical reactions are demonstrated as a result of abundant extracellular enzymes (Sinsabaugh et al., 2008; Kuzyakov and Blagodatskaya, 2015; Li et al., 2019c). Therefore, the linkage between such biotic hotspots and microbial community composition and functioning will be crucial for soil and ecosystem health.

### Microbial Community Composition, Networked Interaction, and Functional Association Enriched in MacA

In the study, the Shannon diversity indices of both bacteria and fungi showed no consistent trend across the aggregate-size fractions or between bacterial and fungal communities, although relatively higher in F-MicA (Table 5). Upton et al. (2019) recently also reported higher Shannon diversity of fungi in MicA than in L-MacA in different bioenergy management systems, although it was subjected to farming management in agricultural soils (Liao et al., 2018; Lu et al., 2018; Li and Yang, 2019). For bacterial community diversity of agricultural soil nonetheless, MicA could be either higher (Zhang et al., 2013; Bach et al., 2018) or lower (Lupwayi et al., 2001). Hereby, the dominant bacterial phyla were generally consistent, whereas fungal phylum composition varied across the size fractions except for the F-Clay fraction. However, based on PERMANOVA (Supplementary Table 4), size of aggregates exerted a significant (*p* < 0.001) but independent impact on both the bacterial and fungal communities of aggregate fractions (Figure 3).

While significantly related to SOC, TN, and C/N ratio (Supplementary Table 5), community composition structures of bacteria in the F-MicA and F-Clay fractions were clearly separated from the L-MacA, MacA, and MicA fractions by NMDS1, whereas that of fungi in L-MacA and MacA were separated by NMDS2, although divergent by NMDS1. In another long-term fertilization study, Li et al. (2019a) also reported a distinct change in fungal and bacterial communities, most closely related to soil carbon and nitrogen contents, in aggregates due to fertilization. This could again suggest that microbial community structure, particularly of fungi, was evidently different in MacA from MicA, particularly in F-MicA, as a dominant pool of soil (Table 2). Again, in this study, the interaction patterns (Figure 4) of bacterial and fungal OTUs, and across bacterial and fungal OTUs, were variable among aggregate-size fractions. While the trends of the positive interactions were roughly similar to the negative interactions among the aggregate-size fractions, bacterial interactions in both positive and negative co-occurrence networks were much more complex and intensive than fungal interactions in each single size fraction. Clearly, all the bacterial, fungal, and bacterial–fungal interactions in MacA (and MicA) were remarkably stronger than in other size fractions, although weak but divergent interactions were detected in the F-MicA and F-Clay fractions. Moreover, the level of co-occurrence network complexity was highest for MacA compared with other fractions. The bacterial communities showed a more compact topology during assembly in MacA and MicA than other fractions, whereas fungal communities showed a looser network, indicating bacterial communities assembled more non-randomly in MacA and MicA whereas fungal communities assembled more randomly (Jiang et al., 2018). Considering that positive correlations indicate synergistic or symbiotic interaction, whereas negative correlations indicate competitive or predatorial interaction between species (Barberan et al., 2012; Faust and Raes, 2012; Lupatini et al., 2014), microbial interactive activity was enhanced in MacA (and MicA), whereas bacterial activity was much higher than fugal activity. Seemingly, the relationships among microbial individuals and the patterns of microbial interaction networks were quite divergent across aggregate-size fractions and were regulated not only by the microbial abundance (Figure 5) and community structure (Figure 4) but also by the specific microenvironment of different soil aggregates, which were raised as concurrent evolutionary incubators for microbial community by Rillig et al. (2017). Being shaped by size of aggregate fractions, the bacterial, fungal, and bacterial–fungal interaction patterns are indeed shifted with macroaggregation. This indicates enhanced microbial community co-occurrence and interaction in MacA rather than in MicA.

As a result of micropore structure of soil with different microenvironments (Tecon and Or, 2017; Juyal et al., 2019; Liang et al., 2019), microbes can interact with each other differently at the microscale (Raynaud and Nunan, 2014). Accordingly, an appropriate distribution of soil pore spaces can regulate the microbial dispersion and functional distribution and even facilitate the interaction within microbiota (Ebrahimi and Or, 2014; Negassa et al., 2015; Juyal et al., 2018; Tecon et al., 2018). Obviously, MicA are mostly occlusive as closely associated with mineral surface and thus can isolate associated microorganisms by spatial exclusion (Six et al., 2000; Lützow et al., 2006; Six and Paustian, 2014). However, the formation of MacA, with MicA occluded and particular organic matter physically protected, increases the complexity in spatial arrangement of soil pores and thus the habitat heterogeneity (Baldock and Skjemstad, 2000). Importantly, microbial interactions, synergistic or competitive, between phyla/species can mediate metabolic functions, affecting the geochemical cycling processes (Wilpiszeski et al., 2019). While Lehmann et al. (2017) noted microbial species interactions across major taxonomic groups in relation to soil aggregation, this study suggests a microscale hotspot of MacA with enhanced microbial community networking and interactions. This further supports our previous finding of enhanced soil enzyme activities in MacA as biochemically functional hotspots in soil matrix (Li et al., 2019c).

Soil aggregation can stabilize microbial composition and strengthen microbial interaction and likely alter microbial community function (Wilpiszeski et al., 2019). In this study, the predictive microbial functional profiles (Tables 5, 6) were altered with the soil aggregate-size fractions of the paddy. Bacterial communities within larger-sized aggregates possessed intense activities in carbon and nitrogen cycling but weak activities involving bacterial migration, DNA metabolism, and biochemical processes for surviving. However, bacterial communities within the smaller-sized fractions, especially F-Clay, were relatively more active in cell motility, DNA metabolism, and intracellular biochemical processes potential for surviving, but less active in assimilation of carbon and nitrogen sources. MacA could be more likely active in functioning of C and N cycling, for ecosystem performance, but those in finer-sized aggregates tended to perform their activity for surviving. This could be related to higher microbial metabolic activities, particularly of C-degrading enzyme activities, found in MacA (Kuzyakov and Blagodatskaya, 2015; Li et al., 2019c) and to the resource limitation in F-MicA due to mineral association and limited pores (Baldock and Skjemstad, 2000), whereas the FUNGuild result (Table 6) indicates that L-MacA tended to be less abundant in guilds of fungal parasite and litter saprotroph than the other fractions except for F-MicA, which was lowest in fungi abundance, but contained more guilds related to disease or fungal parasitic but less of mycorrhizal and dung or saprotrophic fungi for OM decay. Lu et al. (2020) also noted a decrease in some fungal parasite guilds with biochar promoting macroaggregation in a rice soil in an adjacent area. Therefore, it suggests a better microenvironment for soil health and carbon cycling in MacA than smaller-sized aggregates.

Microorganisms adapted to rapid growth depending on easily assimilable substrate and slow growth using relatively recalcitrant substrate are defined, respectively, as r-strategists and K-strategists (Fontaine et al., 2003). r-Strategy is associated with a greater abundance of genes related to regulation and cell signaling, whereas K-strategy is rich in motility and chemotaxis-related genes (Song et al., 2017). Accordingly, the functional profiling here can be translated as that the aggregate fractions in larger size tended to possess both abundant r-strategists and K-strategists. whereas those in smaller size tended to be dominated by K-strategists. In F-MicA and F-Clay particles, diffusion transport of microbial signal molecules can be severely limited at the micrometer scale (Vincent et al., 2010). Instead, larger-sized MacA, MacA, and, to some extent, MicA can provide a balanced combination of substrate availability, diffusion passages, and vicinity to other microbes, allowing active microbial growing and functioning (Nannipieri et al., 2003; Ekschmitt et al., 2005) in addition to microbes with K-strategy in (fine) MicA survived within MacA. Considering their trophic type, microorganisms can also be divided into oligotrophs and copiotrophs associated with, respectively, available and limited resources (Koch, 2001). A copiotrophic strategy is associated with a greater abundance of genes related to cell division and cell cycle, whereas an oligotrophic strategy has a greater abundance of genes related to carbohydrate metabolism and virulence, disease, and defense (Song et al., 2017). Indeed, larger-sized MacA possessed less fungal guilds of fungal parasite and litter saprotroph but higher relative abundance of Basidiomycetes (Figure 2B). As pointed out by Davinic et al. (2012), copiotrophs outcompeted slower growing oligotrophs in L-MacA for abundant labile organic matter in MacA. This is coincident with the higher abundance of functions associated with cell division and cell cycle for rapid growth observed in MacA (Table 5). All these further support MacA as biogeochemically functional hotspots, which was raised in previous studies (Kuzyakov and Blagodatskaya, 2015; Li et al., 2019c).

Overall, this study suggests a coevolution of SOC accumulation and soil bioactivity in rice paddy through the promotion of microbial abundance and activity with the enhancement of physically protected OC in larger-sized MacA under fertilization (Pan et al., 2014, 2015). Likewise, King et al. (2019) commented that reduced MacA turnover can promote SOC accumulation via the stabilization of C into occluded fractions. More importantly, this study further demonstrates the sand-sized MacA as micro-hotspots (Kuzyakov and Blagodatskaya, 2015) with enriched abundance in relation to SOC accumulation and networked interactions for functioning in carbon cycling and also possibly for soil health (Lucas et al., 2014; Vogel et al., 2014). Yet, the linkage of MacA dynamics to plant growth performance and ecosystem health deserves further field studies.

### Microbial Abundance, Community Composition, and Diversity Affected by Fertilization Rather in MacA

In this study, the total variation of microbial abundance (Table 4) and community structure of soil aggregates by NMDS analysis (Figure 3) was shaped by size with a minor impact by fertilization treatment. Using a sonication-wet sieving protocol, Sessitsch et al. (2001) first noted that bacterial populations and community structure were affected rather by the particle size fraction than by the very contrasting organic amendments, in a long-term field experiment from Sweden. Using an “optimal moisture” approach of aggregate isolation, Bach et al. (2018) reported that bacterial and fungal community structure and diversity varied among soil aggregates, regardless of land management of prairie soils. Using a wet-sieving protocol like this study, Davinic et al. (2012) addressed distinct microbial community abundance and structure related to carbon composition between MacA and MicA other than among types of agroecosystems.

However, this study did show microbial community changes among aggregates fractions rather impacted by fertilization than by aggregates size, being generally lower in diversity under CFM compared with NF (Table 5, detailed in Supplementary Table 3). Particularly, fungal diversity exerted clear change with fertilization despite of a generally increasing trend with decreasing size of aggregate fractions. Compared with NF, there was a significant decrease under CFM, an insignificant decrease under CF, and an insignificant increase under CFS, in fungal diversity of aggregates in size larger than 53 μm (Table 5). This decrease in fungal diversity in these larger-sized aggregates under CFM and CF could be attributed to the sensitivity of fungi to heavy metal accumulation of Pb and Cu, particularly in the available pool (Li et al., 2003). A decline in culturable fungal population size and in the ratio of fungal to bacterial PLFAs was observed consistently across the sites of metal contaminated rice paddies across South China (Liu et al., 2012). Again, there was an evident change with treatments in the fungal community composition of MacA compared with MicA in size < 250 μm (Figure 1). In detail, the relative abundance of *Ascomycota* was decreased in L-MacA, whereas that of Basidiomycota and Zygomycota increased, and that of Glomeromycota and unspecified fungal communities markedly decreased both in L-MacA and MacA, under CF and CFM, as compared with NF. In contrast to this study, Yan et al. (2018) reported a decreased relative abundance of Basidiomycota and Zygomycota but increased that of Ascomycota, Chytridiomycota, and Glomeromycota for the whole paddy soil with field contamination of metals. Overall, although diversity cannot be a sensitive indicator for microbial communities among size fractions of aggregates (Lauber et al., 2009), particularly bacterial community herein, diversity and composition with the relative proportion of microbial individuals in MacA are found altered by management practices (Davinic et al., 2012), such as fertilization in this study.

**FIGURE 6:**
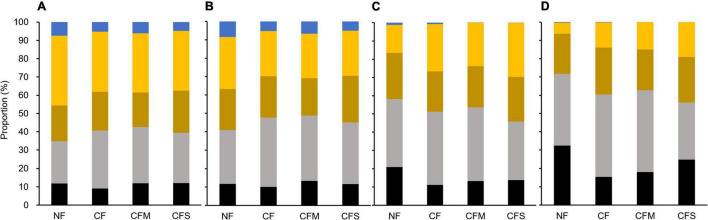
Proportion of mass **(A)**, SOC **(B)**, bacterial abundance **(C)**, and fungal abundance **(D)** by different-size fractions of soil aggregates under the fertilization treatments. Black, L-MacA; gray, MacA; Brown, MicA; yellow, F-MicA; blue, F-Clay. NF, no fertilizer applied; CF, compound inorganic fertilizer; CFM, inorganic fertilizer combined with swine manure; CFS, inorganic fertilizer combined with residue return.

In addition, the proportions by different-size fractions of aggregates among the fertilization treatments are shown in Figure 6. Hereby, compared with mass proportion (Figure 6A), the proportion by MacA became increasingly dominant for SOC (Figure 6B), bacterial (Figure 6C), and fungal abundance (Figure 6D). However, compared with the SOC proportion by L-MacA unchanged with fertilization, the fungal proportion by L-MacA decreased with CF and CFM treatment, although the bacterial proportion was similar among the fertilized plots. This can infer that the enriched fungal population in L-MacA is not favoring macroaggregation possibly due to the stress by metal contamination mentioned previously. Again, MacA functioned as hotspots supporting a dominant portion of microbes, which can be modified to a lesser extent by fertilization. As Nie et al. (2014) advocated, the distribution of aggregates within soils can be affected by both physical and biological processes and in turn affected microbial function in response to climate change. Therefore, fertilization can alter the soil microbial distribution pattern mainly by modifying the relative proportion of different-size fractions, thus affecting the extent of microbial interaction among aggregates shaped by size.

## Conclusion

This study provides general insight into microbial community structure, individual interaction, and functional profile across aggregate-size fractions of a paddy soil under long-term conventional fertilization regimens from the Taihu Plan, China. Our results revealed that SOC and microbial abundance were both enriched in L-MacA and MacA but depleted in F-Clay particles across the treatments. Meanwhile, compared with MicA, microbial abundance concerning SOC accumulation was increased, and microbial networking and interaction were enhanced in MacA, with higher abundances of r-strategists and copiotrophs with more functional genes related to carbon and nitrogen metabolism. Also, soil MacA tended to contain fewer guilds of fungal parasite and litter saprotroph, suggesting better soil health with macroaggregation promoted under combined organic/inorganic fertilization. Thereby, this study confirms MacA as micro-hotspots for microbial community networking, microbial functioning, and potential plant defense. Of course, fertilization can modify the aggregate mass proportion and in turn microbial composition by macroaggregation. Consequently, our study suggests that spatial distribution or isolation of microbes in differently sized soil aggregates can underspin the soil fertility and health changes with fertilization in rice paddy. Moreover, the dynamics of microbial distribution, interaction, and function within aggregate-size fractions can matter soil functions for plant production and ecosystem health in rice paddy under the pressure of food production and climate change.

## Data Availability Statement

The datasets presented in this study can be found in online repositories. The names of the repository/repositories and accession number(s) can be found in the article/Supplementary Material.

## Author Contributions

ZR, GP, ZCL, DZ, and SS designed the research. ZR, ZCL, and GP performed the data analysis. ZR wrote the manuscript. GP, XDL, ZL, HL, XYL, JZ, MD, KC, RB, XZ, and LL revised and commented on the draft. All authors contributed to the article and approved the submitted version.

## Conflict of Interest

The authors declare that the research was conducted in the absence of any commercial or financial relationships that could be construed as a potential conflict of interest.

## Publisher’s Note

All claims expressed in this article are solely those of the authors and do not necessarily represent those of their affiliated organizations, or those of the publisher, the editors and the reviewers. Any product that may be evaluated in this article, or claim that may be made by its manufacturer, is not guaranteed or endorsed by the publisher.

## Footnotes

1
http://hannonlab.cshl.edu/fastx_toolkit

2
http://rdp.cme.msu.edu/

3
http://unite.ut.ee/index.php
