# Supplementary material for: Macroaggregates Serve as Micro-Hotspots Enriched With Functional and Networked Microbial Communities and Enhanced Under Organic/Inorganic Fertilization in a Paddy Topsoil From Southeastern China
Source: Front Microbiol. 2022 Apr 11;13:831746. doi: 10.3389/fmicb.2022.831746 (PMC9039729; doi:10.3389/fmicb.2022.831746)
Supplement: Supplementary file 2 [file Image_2.PDF]

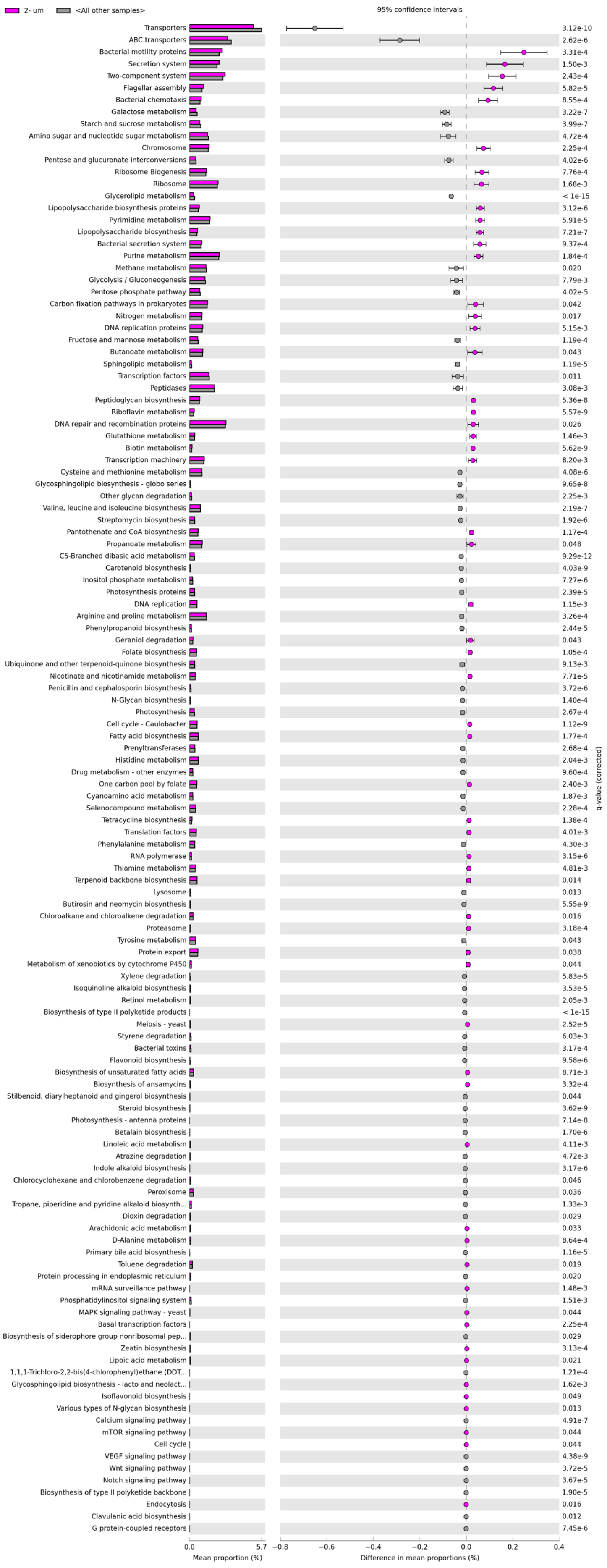
2- um
<All other samples>
95% confidence intervals
Transporters
ABC transporters
Bacterial motility proteins
Secretion system
Two-component system
Flagellar assembly
Bacterial chemotaxis
Galactose metabolism
Starch and sucrose metabolism
Amino sugar and nucleotide sugar metabolism
Chromosome
Pentose and glucuronate interconversions
Ribosome Biogenesis
Ribosome
Glycerolipid metabolism
Lipopolysaccharide biosynthesis proteins
Pyrimidine metabolism
Lipopolysaccharide biosynthesis
Bacterial secretion system
Purine metabolism
Methane metabolism
Glycolysis / Gluconeogenesis
Pentose phosphate pathway
Carbon fixation pathways in prokaryotes
Nitrogen metabolism
DNA replication proteins
Fructose and mannose metabolism
Butanoate metabolism
Sphingolipid metabolism
Transcription factors
Peptidases
Peptidoglycan biosynthesis
Riboflavin metabolism
DNA repair and recombination proteins
Glutathione metabolism
Biotin metabolism
Transcription machinery
Cysteine and methionine metabolism
Glycosphingolipid biosynthesis - globo series
Other glycan degradation
Valine, leucine and isoleucine biosynthesis
Streptomycin biosynthesis
Pantothenate and CoA biosynthesis
Propanoate metabolism
C5-Branched dibasic acid metabolism
Carotenoid biosynthesis
Inositol phosphate metabolism
Photosynthesis proteins
DNA replication
Arginine and proline metabolism
Phenylpropanoid biosynthesis
Geraniol degradation
Folate biosynthesis
Ubiquinone and other terpenoid-quinone biosynthesis
Nicotinate and nicotinamide metabolism
Penicillin and cephalosporin biosynthesis
N-Glycan biosynthesis
Photosynthesis
Cell cycle - Caulobacter
Fatty acid biosynthesis
Prenyltransferases
Histidine metabolism
Drug metabolism - other enzymes
One carbon pool by folate
Cyanoamino acid metabolism
Selenocompound metabolism
Tetracycline biosynthesis
Translation factors
Phenylalanine metabolism
RNA polymerase
Thiamine metabolism
Terpenoid backbone biosynthesis
Lysosome
Butirosin and neomycin biosynthesis
Chloroalkane and chloroalkene degradation
Proteasome
Tyrosine metabolism
Protein export
Metabolism of xenobiotics by cytochrome P450
Xylene degradation
Isoquinoline alkaloid biosynthesis
Retinol metabolism
Biosynthesis of type II polyketide products
Meiosis - yeast
Styrene degradation
Bacterial toxins
Flavonoid biosynthesis
Biosynthesis of unsaturated fatty acids
Biosynthesis of ansamycins
Stilbenoid, diarylheptanoid and gingerol biosynthesis
Steroid biosynthesis
Photosynthesis - antenna proteins
Betalain biosynthesis
Linoleic acid metabolism
Atrazine degradation
Indole alkaloid biosynthesis
Chlorocyclohexane and chlorobenzene degradation
Peroxisome
Tropane, piperidine and pyridine alkaloid biosynth...
Dioxin degradation
Arachidonic acid metabolism
D-Alanine metabolism
Primary bile acid biosynthesis
Toluene degradation
Protein processing in endoplasmic reticulum
mRNA surveillance pathway
Phosphatidylinositol signaling system
MAPK signaling pathway - yeast
Basal transcription factors
Biosynthesis of siderophore group nonribosomal pep...
Zeatin biosynthesis
Lipoic acid metabolism
1,1,1-Trichloro-2,2-bis(4-chlorophenyl)ethane (DDT...
Glycosphingolipid biosynthesis - lacto and neolact...
Isoflavonoid biosynthesis
Various types of N-glycan biosynthesis
Calcium signaling pathway
mTOR signaling pathway
Cell cycle
VEGF signaling pathway
Wnt signaling pathway
Notch signaling pathway
Biosynthesis of type II polyketide backbone
Endocytosis
Clavulanic acid biosynthesis
G protein-coupled receptors
0.0
5.7
Mean proportion (%)
−0.8
−0.6
−0.4
−0.2
0.0
0.2
0.4
Difference in mean proportions (%)
q-value (corrected)
3.12e-10
2.62e-6
3.31e-4
1.50e-3
2.43e-4
5.82e-5
8.55e-4
3.22e-7
3.99e-7
4.72e-4
2.25e-4
4.02e-6
7.76e-4
1.68e-3
< 1e-15
3.12e-6
5.91e-5
7.21e-7
9.37e-4
1.84e-4
0.020
7.79e-3
4.02e-5
0.042
0.017
5.15e-3
1.19e-4
0.043
1.19e-5
0.011
3.08e-3
5.36e-8
5.57e-9
0.026
1.46e-3
5.62e-9
8.20e-3
4.08e-6
9.65e-8
2.25e-3
2.19e-7
1.92e-6
1.17e-4
0.048
9.29e-12
4.03e-9
7.27e-6
2.39e-5
1.15e-3
3.26e-4
2.44e-5
0.043
1.05e-4
9.13e-3
7.71e-5
3.72e-6
1.40e-4
2.67e-4
1.12e-9
1.77e-4
2.68e-4
2.04e-3
9.60e-4
2.40e-3
1.87e-3
2.28e-4
1.38e-4
4.01e-3
4.30e-3
3.15e-6
4.81e-3
0.014
0.013
5.55e-9
0.016
3.18e-4
0.043
0.038
0.044
5.83e-5
3.53e-5
2.05e-3
< 1e-15
2.52e-5
6.03e-3
3.17e-4
9.58e-6
8.71e-3
3.32e-4
0.044
3.62e-9
7.14e-8
1.70e-6
4.11e-3
4.72e-3
3.17e-6
0.046
0.036
1.33e-3
0.029
0.033
8.64e-4
1.16e-5
0.019
0.020
1.48e-3
1.51e-3
0.044
2.25e-4
0.029
3.13e-4
0.021
1.21e-4
1.62e-3
0.049
0.013
4.91e-7
0.044
0.044
4.38e-9
3.72e-5
3.67e-5
1.90e-5
0.016
0.012
7.45e-6

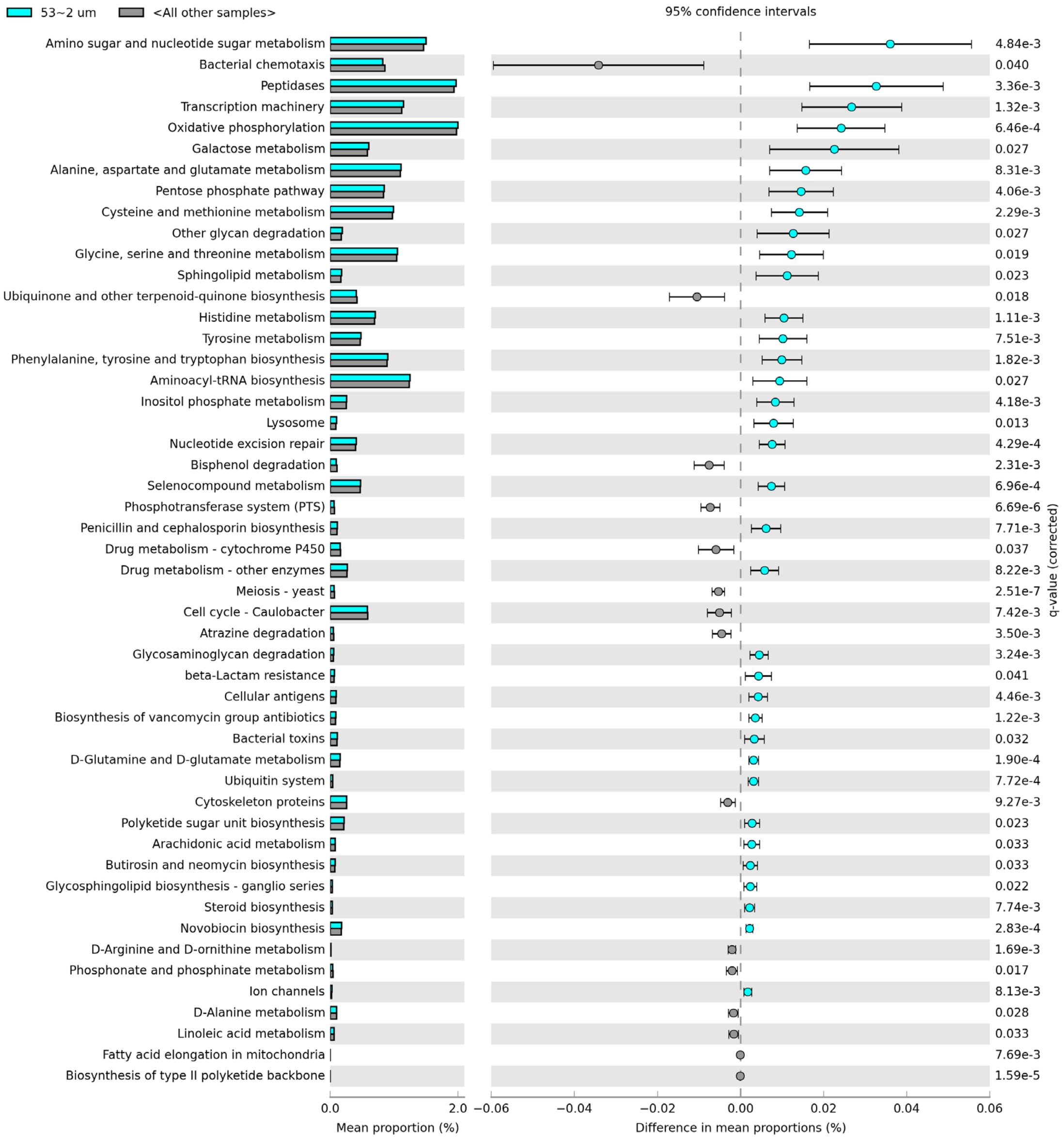
53~2 um
<All other samples>
95% confidence intervals
Amino sugar and nucleotide sugar metabolism
Bacterial chemotaxis
Peptidases
Transcription machinery
Oxidative phosphorylation
Galactose metabolism
Alanine, aspartate and glutamate metabolism
Pentose phosphate pathway
Cysteine and methionine metabolism
Other glycan degradation
Glycine, serine and threonine metabolism
Sphingolipid metabolism
Ubiquinone and other terpenoid-quinone biosynthesis
Histidine metabolism
Tyrosine metabolism
Phenylalanine, tyrosine and tryptophan biosynthesis
Aminoacyl-tRNA biosynthesis
Inositol phosphate metabolism
Lysosome
Nucleotide excision repair
Bisphenol degradation
Selenocompound metabolism
Phosphotransferase system (PTS)
Penicillin and cephalosporin biosynthesis
Drug metabolism - cytochrome P450
Drug metabolism - other enzymes
Meiosis - yeast
Cell cycle - Caulobacter
Atrazine degradation
Glycosaminoglycan degradation
beta-Lactam resistance
Cellular antigens
Biosynthesis of vancomycin group antibiotics
Bacterial toxins
D-Glutamine and D-glutamate metabolism
Ubiquitin system
Cytoskeleton proteins
Polyketide sugar unit biosynthesis
Arachidonic acid metabolism
Butirosin and neomycin biosynthesis
Glycosphingolipid biosynthesis - ganglio series
Steroid biosynthesis
Novobiocin biosynthesis
D-Arginine and D-ornithine metabolism
Phosphonate and phosphinate metabolism
Ion channels
D-Alanine metabolism
Linoleic acid metabolism
Fatty acid elongation in mitochondria
Biosynthesis of type II polyketide backbone
4.84e-3
0.040
3.36e-3
1.32e-3
6.46e-4
0.027
8.31e-3
4.06e-3
2.29e-3
0.027
0.019
0.023
0.018
1.11e-3
7.51e-3
1.82e-3
0.027
4.18e-3
0.013
4.29e-4
2.31e-3
6.96e-4
6.69e-6
7.71e-3
0.037
8.22e-3
2.51e-7
7.42e-3
3.50e-3
3.24e-3
0.041
4.46e-3
1.22e-3
0.032
1.90e-4
7.72e-4
9.27e-3
0.023
0.033
0.033
0.022
7.74e-3
2.83e-4
1.69e-3
0.017
8.13e-3
0.028
0.033
7.69e-3
1.59e-5
q-value (corrected)
0.0
2.0
−0.06
−0.04
−0.02
0.00
0.02
0.04
0.06
Mean proportion (%)
Difference in mean proportions (%)

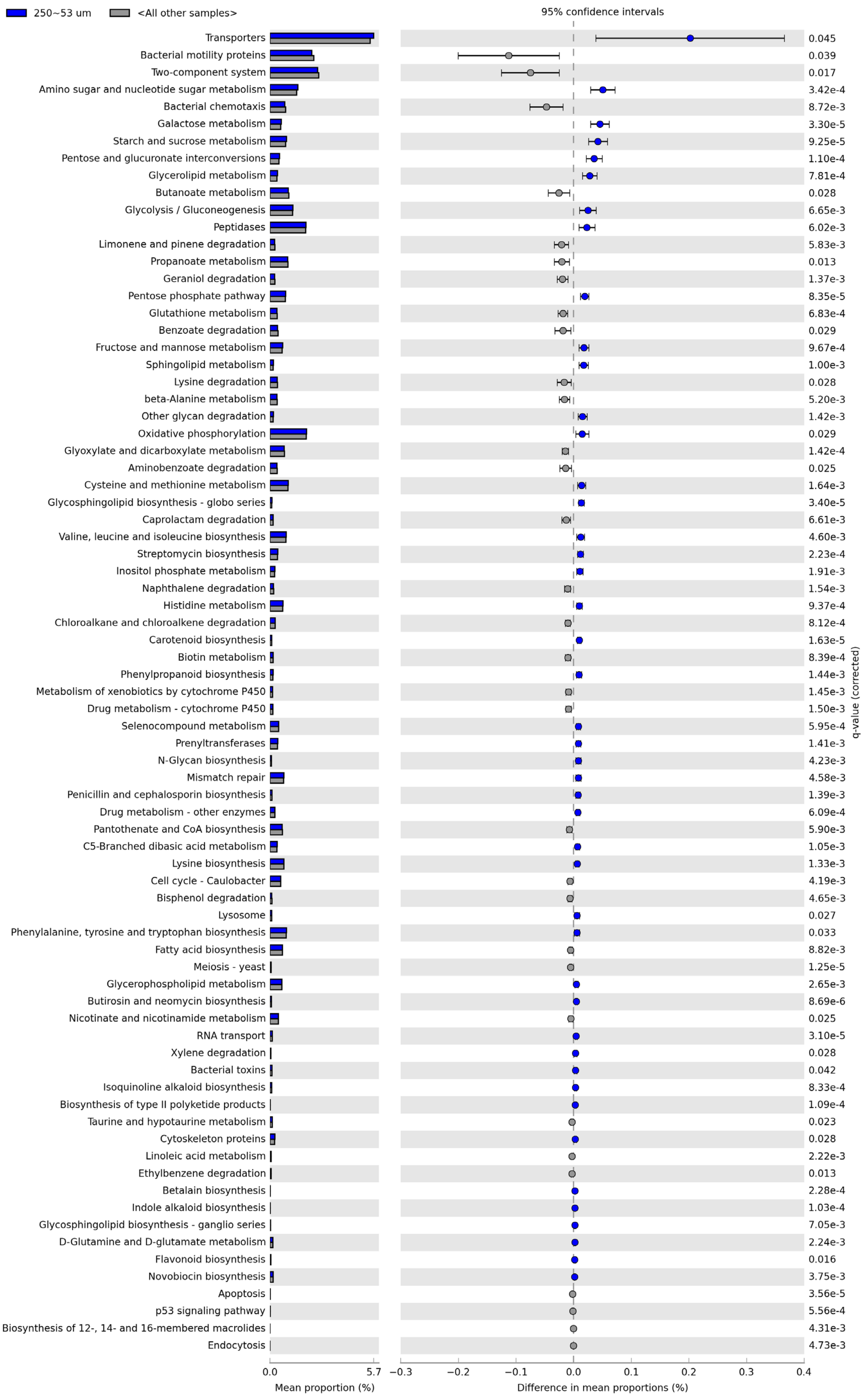
250~53 um
<All other samples>
95% confidence intervals
Transporters 0.045
Bacterial motility proteins 0.039
Two-component system 0.017
Amino sugar and nucleotide sugar metabolism 3.42e-4
Bacterial chemotaxis 8.72e-3
Galactose metabolism 3.30e-5
Starch and sucrose metabolism 9.25e-5
Pentose and glucuronate interconversions 1.10e-4
Glycerolipid metabolism 7.81e-4
Butanoate metabolism 0.028
Glycolysis / Gluconeogenesis 6.65e-3
Peptidases 6.02e-3
Limonene and pinene degradation 5.83e-3
Propanoate metabolism 0.013
Geraniol degradation 1.37e-3
Pentose phosphate pathway 8.35e-5
Glutathione metabolism 6.83e-4
Benzoate degradation 0.029
Fructose and mannose metabolism 9.67e-4
Sphingolipid metabolism 1.00e-3
Lysine degradation 0.028
beta-Alanine metabolism 5.20e-3
Other glycan degradation 1.42e-3
Oxidative phosphorylation 0.029
Glyoxylate and dicarboxylate metabolism 1.42e-4
Aminobenzoate degradation 0.025
Cysteine and methionine metabolism 1.64e-3
Glycosphingolipid biosynthesis - globo series 3.40e-5
Caprolactam degradation 6.61e-3
Valine, leucine and isoleucine biosynthesis 4.60e-3
Streptomycin biosynthesis 2.23e-4
Inositol phosphate metabolism 1.91e-3
Naphthalene degradation 1.54e-3
Histidine metabolism 9.37e-4
Chloroalkane and chloroalkene degradation 8.12e-4
Carotenoid biosynthesis 1.63e-5
Biotin metabolism 8.39e-4
Phenylpropanoid biosynthesis 1.44e-3
Metabolism of xenobiotics by cytochrome P450 1.45e-3
Drug metabolism - cytochrome P450 1.50e-3
Selenocompound metabolism 5.95e-4
Prenyltransferases 1.41e-3
N-Glycan biosynthesis 4.23e-3
Mismatch repair 4.58e-3
Penicillin and cephalosporin biosynthesis 1.39e-3
Drug metabolism - other enzymes 6.09e-4
Pantothenate and CoA biosynthesis 5.90e-3
C5-Branched dibasic acid metabolism 1.05e-3
Lysine biosynthesis 1.33e-3
Cell cycle - Caulobacter 4.19e-3
Bisphenol degradation 4.65e-3
Lysosome 0.027
Phenylalanine, tyrosine and tryptophan biosynthesis 0.033
Fatty acid biosynthesis 8.82e-3
Meiosis - yeast 1.25e-5
Glycerophospholipid metabolism 2.65e-3
Butirosin and neomycin biosynthesis 8.69e-6
Nicotinate and nicotinamide metabolism 0.025
RNA transport 3.10e-5
Xylene degradation 0.028
Bacterial toxins 0.042
Isoquinoline alkaloid biosynthesis 8.33e-4
Biosynthesis of type II polyketide products 1.09e-4
Taurine and hypotaurine metabolism 0.023
Cytoskeleton proteins 0.028
Linoleic acid metabolism 2.22e-3
Ethylbenzene degradation 0.013
Betalain biosynthesis 2.28e-4
Indole alkaloid biosynthesis 1.03e-4
Glycosphingolipid biosynthesis - ganglio series 7.05e-3
D-Glutamine and D-glutamate metabolism 2.24e-3
Flavonoid biosynthesis 0.016
Novobiocin biosynthesis 3.75e-3
Apoptosis 3.56e-5
p53 signaling pathway 5.56e-4
Biosynthesis of 12-, 14- and 16-membered macrolides 4.31e-3
Endocytosis 4.73e-3
q-value (corrected)
0.0
5.7
Mean proportion (%)
−0.3
−0.2
−0.1
0.0
0.1
0.2
0.3
0.4
Difference in mean proportions (%)

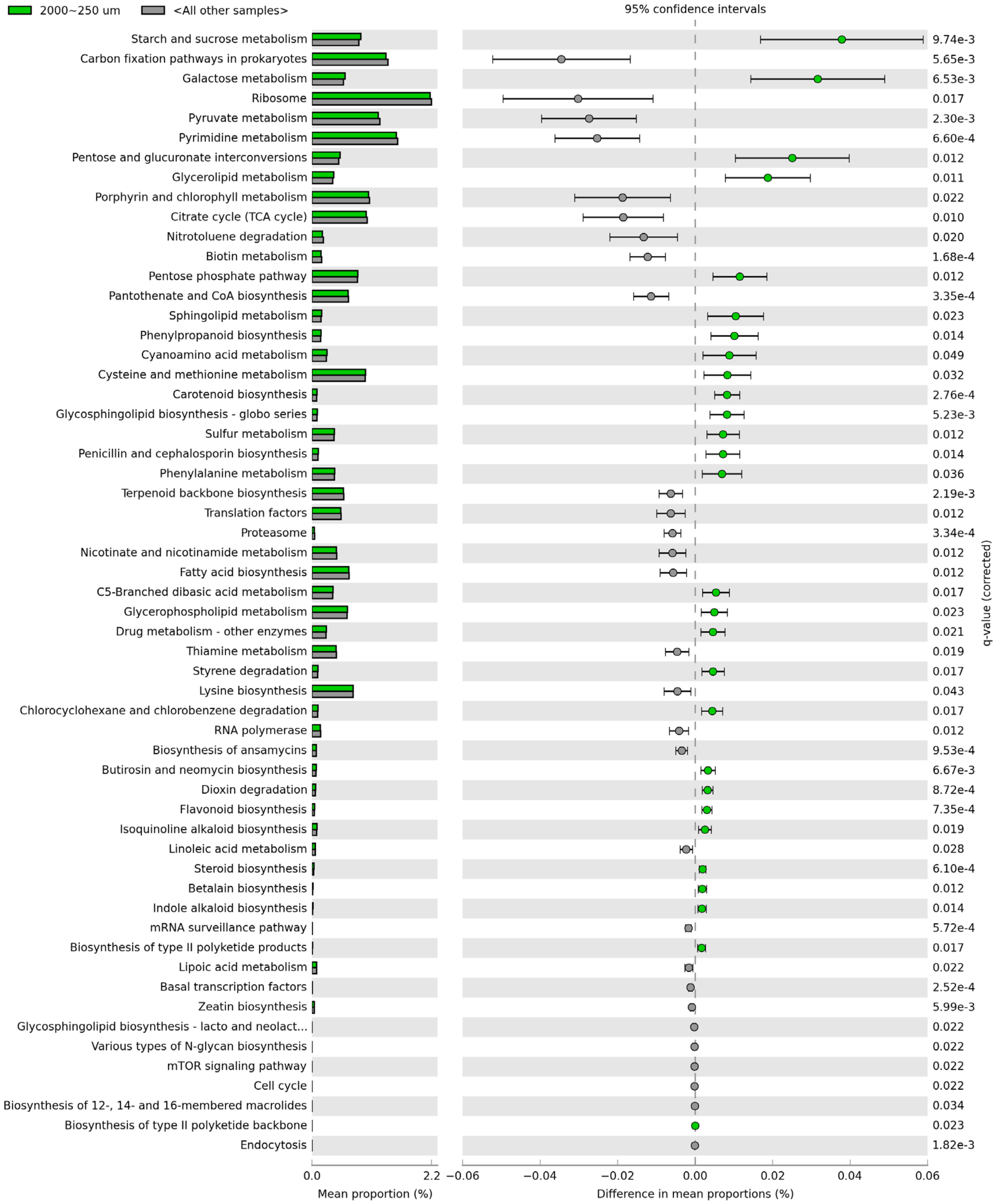
2000~250 um
<All other samples>
95% confidence intervals
Starch and sucrose metabolism 9.74e-3
Carbon fixation pathways in prokaryotes 5.65e-3
Galactose metabolism 6.53e-3
Ribosome 0.017
Pyruvate metabolism 2.30e-3
Pyrimidine metabolism 6.60e-4
Pentose and glucuronate interconversions 0.012
Glycerolipid metabolism 0.011
Porphyrin and chlorophyll metabolism 0.022
Citrate cycle (TCA cycle) 0.010
Nitrotoluene degradation 0.020
Biotin metabolism 1.68e-4
Pentose phosphate pathway 0.012
Pantothenate and CoA biosynthesis 3.35e-4
Sphingolipid metabolism 0.023
Phenylpropanoid biosynthesis 0.014
Cyanoamino acid metabolism 0.049
Cysteine and methionine metabolism 0.032
Carotenoid biosynthesis 2.76e-4
Glycosphingolipid biosynthesis - globo series 5.23e-3
Sulfur metabolism 0.012
Penicillin and cephalosporin biosynthesis 0.014
Phenylalanine metabolism 0.036
Terpenoid backbone biosynthesis 2.19e-3
Translation factors 0.012
Proteasome 3.34e-4
Nicotinate and nicotinamide metabolism 0.012
Fatty acid biosynthesis 0.012
C5-Branched dibasic acid metabolism 0.017
Glycerophospholipid metabolism 0.023
Drug metabolism - other enzymes 0.021
Thiamine metabolism 0.019
Styrene degradation 0.017
Lysine biosynthesis 0.043
Chlorocyclohexane and chlorobenzene degradation 0.017
RNA polymerase 0.012
Biosynthesis of ansamycins 9.53e-4
Butirosin and neomycin biosynthesis 6.67e-3
Dioxin degradation 8.72e-4
Flavonoid biosynthesis 7.35e-4
Isoquinoline alkaloid biosynthesis 0.019
Linoleic acid metabolism 0.028
Steroid biosynthesis 6.10e-4
Betalain biosynthesis 0.012
Indole alkaloid biosynthesis 0.014
mRNA surveillance pathway 5.72e-4
Biosynthesis of type II polyketide products 0.017
Lipoic acid metabolism 0.022
Basal transcription factors 2.52e-4
Zeatin biosynthesis 5.99e-3
Glycosphingolipid biosynthesis - lacto and neolact... 0.022
Various types of N-glycan biosynthesis 0.022
mTOR signaling pathway 0.022
Cell cycle 0.022
Biosynthesis of 12-, 14- and 16-membered macrolides 0.034
Biosynthesis of type II polyketide backbone 0.023
Endocytosis 1.82e-3
0.0
2.2
Mean proportion (%)
−0.06
−0.04
−0.02
0.00
0.02
0.04
0.06
Difference in mean proportions (%)
q-value (corrected)

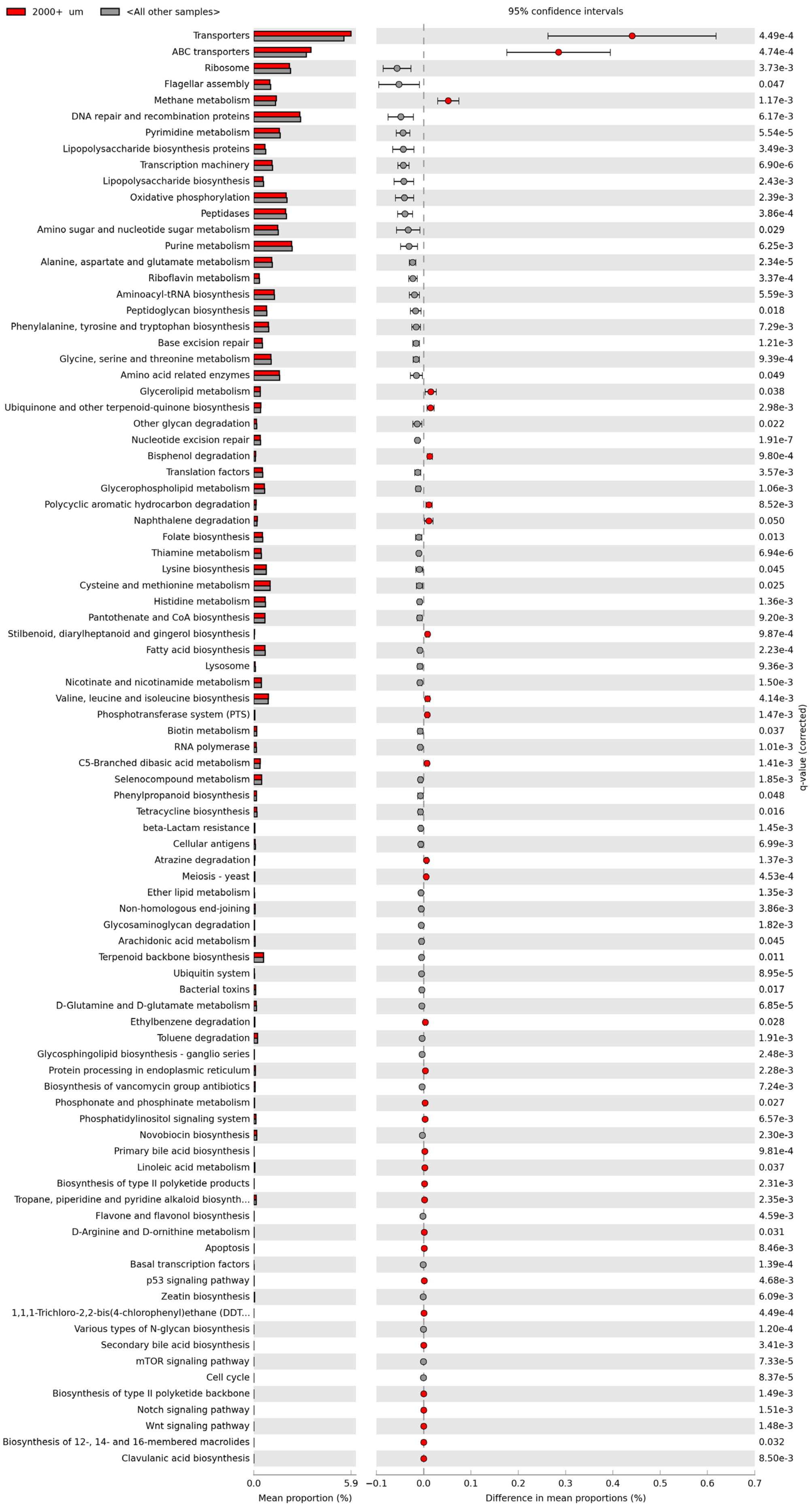

2000+ um
<All other samples>
95% confidence intervals
Transporters
ABC transporters
Ribosome
Flagellar assembly
Methane metabolism
DNA repair and recombination proteins
Pyrimidine metabolism
Lipopolysaccharide biosynthesis proteins
Transcription machinery
Lipopolysaccharide biosynthesis
Oxidative phosphorylation
Peptidases
Amino sugar and nucleotide sugar metabolism
Purine metabolism
Alanine, aspartate and glutamate metabolism
Riboflavin metabolism
Aminoacyl-tRNA biosynthesis
Peptidoglycan biosynthesis
Phenylalanine, tyrosine and tryptophan biosynthesis
Base excision repair
Glycine, serine and threonine metabolism
Amino acid related enzymes
Glycerolipid metabolism
Ubiquinone and other terpenoid-quinone biosynthesis
Other glycan degradation
Nucleotide excision repair
Bisphenol degradation
Translation factors
Glycerophospholipid metabolism
Polycyclic aromatic hydrocarbon degradation
Naphthalene degradation
Folate biosynthesis
Thiamine metabolism
Lysine biosynthesis
Cysteine and methionine metabolism
Histidine metabolism
Pantothenate and CoA biosynthesis
Stilbenoid, diarylheptanoid and gingerol biosynthesis
Fatty acid biosynthesis
Lysosome
Nicotinate and nicotinamide metabolism
Valine, leucine and isoleucine biosynthesis
Phosphotransferase system (PTS)
Biotin metabolism
RNA polymerase
C5-Branched dibasic acid metabolism
Selenocompound metabolism
Phenylpropanoid biosynthesis
Tetracycline biosynthesis
beta-Lactam resistance
Cellular antigens
Atrazine degradation
Meiosis - yeast
Ether lipid metabolism
Non-homologous end-joining
Glycosaminoglycan degradation
Arachidonic acid metabolism
Terpenoid backbone biosynthesis
Ubiquitin system
Bacterial toxins
D-Glutamine and D-glutamate metabolism
Ethylbenzene degradation
Toluene degradation
Glycosphingolipid biosynthesis - ganglio series
Protein processing in endoplasmic reticulum
Biosynthesis of vancomycin group antibiotics
Phosphonate and phosphinate metabolism
Phosphatidylinositol signaling system
Novobiocin biosynthesis
Primary bile acid biosynthesis
Linoleic acid metabolism
Biosynthesis of type II polyketide products
Tropane, piperidine and pyridine alkaloid biosynth...
Flavone and flavonol biosynthesis
D-Arginine and D-ornithine metabolism
Apoptosis
Basal transcription factors
p53 signaling pathway
Zeatin biosynthesis
1,1,1-Trichloro-2,2-bis(4-chlorophenyl)ethane (DDT...
Various types of N-glycan biosynthesis
Secondary bile acid biosynthesis
mTOR signaling pathway
Cell cycle
Biosynthesis of type II polyketide backbone
Notch signaling pathway
Wnt signaling pathway
Biosynthesis of 12-, 14- and 16-membered macrolides
Clavulanic acid biosynthesis
4.49e-4
4.74e-4
3.73e-3
0.047
1.17e-3
6.17e-3
5.54e-5
3.49e-3
6.90e-6
2.43e-3
2.39e-3
3.86e-4
0.029
6.25e-3
2.34e-5
3.37e-4
5.59e-3
0.018
7.29e-3
1.21e-3
9.39e-4
0.049
0.038
2.98e-3
0.022
1.91e-7
9.80e-4
3.57e-3
1.06e-3
8.52e-3
0.050
0.013
6.94e-6
0.045
0.025
1.36e-3
9.20e-3
9.87e-4
2.23e-4
9.36e-3
1.50e-3
4.14e-3
1.47e-3
0.037
1.01e-3
1.41e-3
1.85e-3
0.048
0.016
1.45e-3
6.99e-3
1.37e-3
4.53e-4
1.35e-3
3.86e-3
1.82e-3
0.045
0.011
8.95e-5
0.017
6.85e-5
0.028
1.91e-3
2.48e-3
2.28e-3
7.24e-3
0.027
6.57e-3
2.30e-3
9.81e-4
0.037
2.31e-3
2.35e-3
4.59e-3
0.031
8.46e-3
1.39e-4
4.68e-3
6.09e-3
4.49e-4
1.20e-4
3.41e-3
7.33e-5
8.37e-5
1.49e-3
1.51e-3
1.48e-3
0.032
8.50e-3
q-value (corrected)
0.0
5.9
Mean proportion (%)
−0.1
0.0
0.1
0.2
0.3
0.4
0.5
0.6
0.7
Difference in mean proportions (%)

SUPPLEMENTARY FIGURE 2 Differences in KEGG pathways of each sized fraction of aggregate samples compared to all the other aggregate samples. Differences were compared with statistical method of two-sided Welch's t-test with 0.95 CI and Benjamini-Hochberg FDR for multiple test, and plotted with features at q-value < 0.05 in the software STAMP.
