# Supplementary material for: Macroaggregates Serve as Micro-Hotspots Enriched With Functional and Networked Microbial Communities and Enhanced Under Organic/Inorganic Fertilization in a Paddy Topsoil From Southeastern China
Source: Front Microbiol. 2022 Apr 11;13:831746. doi: 10.3389/fmicb.2022.831746 (PMC9039729; doi:10.3389/fmicb.2022.831746)
Supplement: Supplementary file 3 [file Image_3.PDF]

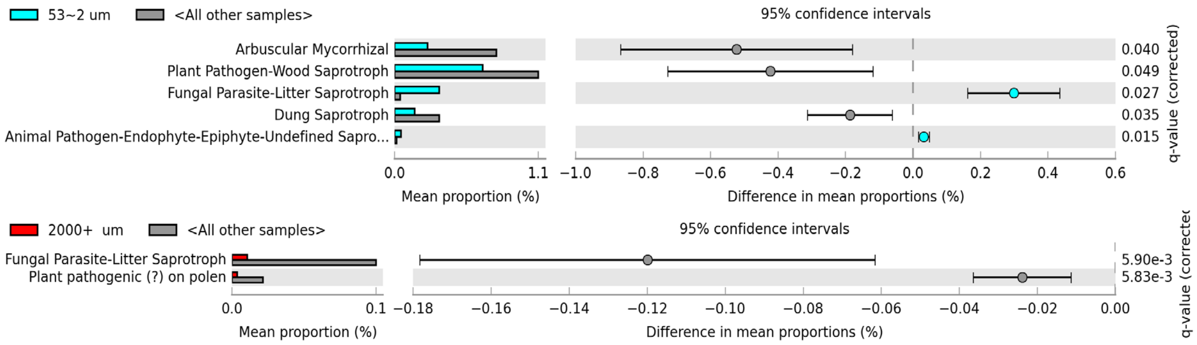

SUPPLEMENTARY FIGURE 3 Differences in fungal functional guilds of each sized fraction of aggregate samples compared to all the other aggregate samples. Differences were compared with statistical method of two-sided Welch's t-test with 0.95 CI and Benjamini-Hochberg FDR for multiple test, and plotted with features at *q*-value < 0.05 in the software STAMP.
