# Supplementary material for: Macroaggregates Serve as Micro-Hotspots Enriched With Functional and Networked Microbial Communities and Enhanced Under Organic/Inorganic Fertilization in a Paddy Topsoil From Southeastern China
Source: Front Microbiol. 2022 Apr 11;13:831746. doi: 10.3389/fmicb.2022.831746 (PMC9039729; doi:10.3389/fmicb.2022.831746)
Supplement: Supplementary file 4 [file Table_1.DOCX]

SUPPLEMENTARY TABLE 1 Two-way ANOVA of basic properties of aggregate size fractions.

|  | Mass Proportion | |  | SOC | |  | TN | |  | C/N ratio | |
| --- | --- | --- | --- | --- | --- | --- | --- | --- | --- | --- | --- |
|  | % of total variation | P value summary |  | % of total variation | P value summary |  | % of total variation | P value summary |  | % of total variation | P value summary |
| Treatment | 0.00 | ns |  | 9.68 | **** |  | 11.96 | *** |  | 1.53 | ns |
| Aggregate | 93.79 | **** |  | 76.09 | **** |  | 62.63 | **** |  | 83.13 | **** |
| Treatment*Aggregate | 3.91 | **** |  | 5.46 | * |  | 6.44 | ns |  | 4.42 | ns |

*, **, *** and **** indicate significances at *P* < 0.05, *P* < 0.01, *P* < 0.001 and *P* < 0.0001, respectively.
