# Supplementary material for: Macroaggregates Serve as Micro-Hotspots Enriched With Functional and Networked Microbial Communities and Enhanced Under Organic/Inorganic Fertilization in a Paddy Topsoil From Southeastern China
Source: Front Microbiol. 2022 Apr 11;13:831746. doi: 10.3389/fmicb.2022.831746 (PMC9039729; doi:10.3389/fmicb.2022.831746)
Supplement: Supplementary file 5 [file Table_2.DOCX]

SUPPLEMENTARY TABLE 2 Two-way ANOVA of bacterial and fungal abundance and B/F ratio of aggregate size fractions.

|  | Bacterial Abundance | |  | Fungal Abundance | |  | B/F ratio | |
| --- | --- | --- | --- | --- | --- | --- | --- | --- |
|  | % of total variation | P value summary |  | % of total variation | P value summary |  | % of total variation | P value summary |
| Treatment | 4.00 | *** |  | 6.52 | ** |  | 7.36 | *** |
| Aggregate | 83.34 | **** |  | 73.52 | **** |  | 64.12 | **** |
| Treatment*Aggregate | 5.36 | * |  | 4.09 | ns |  | 15.01 | *** |

*, **, *** and **** indicate significances at *P* < 0.05, *P* < 0.01, *P* < 0.001 and *P* < 0.0001, respectively.
