# Supplementary material for: Macroaggregates Serve as Micro-Hotspots Enriched With Functional and Networked Microbial Communities and Enhanced Under Organic/Inorganic Fertilization in a Paddy Topsoil From Southeastern China
Source: Front Microbiol. 2022 Apr 11;13:831746. doi: 10.3389/fmicb.2022.831746 (PMC9039729; doi:10.3389/fmicb.2022.831746)
Supplement: Supplementary file 6 [file Table_3.DOCX]

SUPPLEMENTARY TABLE 3 Two-way ANOVA of Shannon index (H) of bacterial and fungal communities in aggregate size fractions.

|  | Bacterial community | |  | Fungal community | |
| --- | --- | --- | --- | --- | --- |
|  | % of total variation | P value summary |  | % of total variation | P value summary |
| Treatment | 41.90 | **** |  | 61.36 | **** |
| Aggregate | 7.46 | ns |  | 14.58 | *** |
| Treatment*Aggregate | 16.48 | ns |  | 4.1 | ns |

Symbols *, **, *** and **** indicate significance values of *P* < 0.05, *P* < 0.01, *P* < 0.001 and *P* < 0.0001, respectively.
