# Supplementary material for: Macroaggregates Serve as Micro-Hotspots Enriched With Functional and Networked Microbial Communities and Enhanced Under Organic/Inorganic Fertilization in a Paddy Topsoil From Southeastern China
Source: Front Microbiol. 2022 Apr 11;13:831746. doi: 10.3389/fmicb.2022.831746 (PMC9039729; doi:10.3389/fmicb.2022.831746)
Supplement: Supplementary file 7 [file Table_4.DOCX]

SUPPLEMENTARY TABLE 4 Permutational multivariate analyses of variance (PERMANOVA) using the Bray-Curtis distance matrices from the bacterial and fungal OTU tables.

|  | Df | Sums Of Sqs | Mean Sqs | F. Model | R^2^ | Pr (>F) |
| --- | --- | --- | --- | --- | --- | --- |
| Bacterial community | | | | | | |
| Aggregate | 4 | 2.13 | 0.53 | 13.20 | 0.38 | 0.001 *** |
| Treatment | 3 | 1.41 | 0.47 | 11.70 | 0.25 | 0.001 *** |
| Aggregate:Treatment | 12 | 0.51 | 0.04 | 1.05 | 0.09 | 0.344 |
| Residuals | 40 | 1.61 | 0.04 |  | 0.28 |  |
| Total | 59 | 5.66 |  |  | 1.00 |  |
| Fungal community | | | | | | |
| Aggregate | 3 | 1.34 | 0.45 | 3.50 | 0.14 | 0.001 *** |
| Treatment | 3 | 3.63 | 1.21 | 9.51 | 0.39 | 0.001 *** |
| Aggregate:Treatment | 9 | 0.77 | 0.09 | 0.67 | 0.08 | 0.997 |
| Residuals | 29 | 3.69 | 0.13 |  | 0.39 |  |
| Total | 44 | 9.43 |  |  | 1.00 |  |

Number of permutations is 999. Symbols *, ** and *** indicate significance values of *P* < 0.05, *P* < 0.01 and *P* < 0.001, respectively.
