# Supplementary material for: Macroaggregates Serve as Micro-Hotspots Enriched With Functional and Networked Microbial Communities and Enhanced Under Organic/Inorganic Fertilization in a Paddy Topsoil From Southeastern China
Source: Front Microbiol. 2022 Apr 11;13:831746. doi: 10.3389/fmicb.2022.831746 (PMC9039729; doi:10.3389/fmicb.2022.831746)
Supplement: Supplementary file 8 [file Table_5.DOCX]

SUPPLEMENTARY TABLE 5 Statistical results of environmental factors fitting onto the NMDS ordinations of the microbial communities.

|  | Bacterial community | |  | Fungal community | |
| --- | --- | --- | --- | --- | --- |
|  | r^2^ | Pr (>r) |  | r^2^ | Pr (>r) |
| SOC | 0.10 | 0.049 * |  | 0.13 | 0.047 * |
| TN | 0.17 | 0.008 ** |  | 0.12 | 0.055 |
| C/N Ratio | 0.39 | 0.001 *** |  | 0.06 | 0.296 |

Calculated by the *envfit* function in the *vegan* package with 999 permutations. Symbols *, ** and *** indicate significance values of *P* < 0.05, *P* < 0.01 and *P* < 0.001, respectively.
