# Supplementary material for: Macroaggregates Serve as Micro-Hotspots Enriched With Functional and Networked Microbial Communities and Enhanced Under Organic/Inorganic Fertilization in a Paddy Topsoil From Southeastern China
Source: Front Microbiol. 2022 Apr 11;13:831746. doi: 10.3389/fmicb.2022.831746 (PMC9039729; doi:10.3389/fmicb.2022.831746)
Supplement: Supplementary file 9 [file Table_6.DOCX]

SUPPLEMENTARY TABLE 6 Topological features of positive co-occurrence networks among size fractions of bacterial consortia, fungal consortia and bacterial-fungal consortia calculated by *igraph* package in R.

|  | Aggregate | Nn | Ne | AD | APL | Den | Mod | Nc | CoC |
| --- | --- | --- | --- | --- | --- | --- | --- | --- | --- |
| Bacterial | <2 μm | 232 | 355 | 3.06 | 4.49 | 0.0132 | 0.76 | 35 | 0.38 |
|  | 53~2 μm | 320 | 783 | 4.89 | 4.03 | 0.0153 | 0.58 | 43 | 0.47 |
|  | 250~53 μm | 394 | 2043 | 10.37 | 3.59 | 0.0264 | 0.43 | 38 | 0.63 |
|  | 2000~250 μm | 412 | 2208 | 10.72 | 3.15 | 0.0261 | 0.46 | 38 | 0.53 |
|  | >2000 μm | 212 | 306 | 2.89 | 3.80 | 0.0137 | 0.73 | 44 | 0.44 |
| Fungal | 53~2 μm | 25 | 16 | 1.28 | 1.11 | 0.0533 | 0.85 | 11 | 0.67 |
|  | 250~53 μm | 76 | 101 | 2.66 | 3.61 | 0.0354 | 0.68 | 12 | 0.39 |
|  | 2000~250 μm | 68 | 67 | 1.97 | 2.49 | 0.0294 | 0.72 | 20 | 0.39 |
|  | >2000 μm | 57 | 56 | 1.96 | 2.71 | 0.0351 | 0.76 | 16 | 0.41 |
| Bacterial-Fungal | 53~2 μm | 50 | 29 | 1.16 | 1.29 | 0.0237 | 0.94 | 21 |  |
|  | 250~53 μm | 269 | 461 | 3.43 | 4.00 | 0.0128 | 0.60 | 30 |  |
|  | 2000~250 μm | 250 | 436 | 3.49 | 3.66 | 0.0140 | 0.56 | 24 |  |
|  | >2000 μm | 106 | 98 | 1.85 | 2.94 | 0.0176 | 0.76 | 30 |  |

Nn, number of nodes; Ne, number of edges; AD, average degree; APL, average path length; Den, density; Mod, modularity; Nc, number of clusters; CoC, clustering coefficient.
