# Supplementary material for: Macroaggregates Serve as Micro-Hotspots Enriched With Functional and Networked Microbial Communities and Enhanced Under Organic/Inorganic Fertilization in a Paddy Topsoil From Southeastern China
Source: Front Microbiol. 2022 Apr 11;13:831746. doi: 10.3389/fmicb.2022.831746 (PMC9039729; doi:10.3389/fmicb.2022.831746)
Supplement: Supplementary file 10 [file Table_7.DOCX]

SUPPLEMENTARY TABLE 7 Topological features of negative co-occurrence networks among size fractions of bacterial consortia, fungal consortia and bacterial-fungal consortia calculated by *igraph* package in R.

|  | Aggregate | Nn | Ne | AD | APL | Den | Mod | Nc |
| --- | --- | --- | --- | --- | --- | --- | --- | --- |
| Bacterial | <2 μm | 144 | 192 | 2.67 | 4.18 | 0.0186 | 0.62 | 20 |
|  | 53~2 μm | 236 | 517 | 4.38 | 4.39 | 0.0186 | 0.49 | 25 |
|  | 250~53 μm | 303 | 1435 | 9.47 | 3.55 | 0.0314 | 0.28 | 26 |
|  | 2000~250 μm | 380 | 1809 | 9.52 | 3.93 | 0.0251 | 0.28 | 33 |
|  | >2000 μm | 159 | 198 | 2.49 | 4.15 | 0.0158 | 0.65 | 30 |
| Fungal | 53~2 μm | 8 | 4 | 1.00 | 1.00 | 0.1429 | 0.75 | 4 |
|  | 250~53 μm | 28 | 23 | 1.64 | 2.81 | 0.0608 | 0.68 | 6 |
|  | 2000~250 μm | 34 | 26 | 1.53 | 2.48 | 0.0463 | 0.74 | 9 |
|  | >2000 μm | 22 | 14 | 1.27 | 1.33 | 0.0606 | 0.86 | 8 |
| Bacterial-Fungal | 53~2 μm | 35 | 21 | 1.20 | 1.29 | 0.0353 | 0.90 | 15 |
|  | 250~53 μm | 218 | 276 | 2.53 | 4.28 | 0.0117 | 0.73 | 27 |
|  | 2000~250 μm | 211 | 313 | 2.97 | 3.62 | 0.0141 | 0.66 | 24 |
|  | >2000 μm | 58 | 47 | 1.62 | 3.28 | 0.0284 | 0.74 | 15 |

Nn, number of nodes; Ne, number of edges; AD, average degree; APL, average path length; Den, density; Mod, modularity; Nc, number of clusters.
